# Trazodone regulates neurotrophic/growth factors, mitogen-activated protein kinases and lactate release in human primary astrocytes

**DOI:** 10.1186/s12974-015-0446-x

**Published:** 2015-12-01

**Authors:** Simona Daniele, Elisa Zappelli, Claudia Martini

**Affiliations:** Department of Pharmacy, University of Pisa, Via Bonanno Pisano, 6, Pisa, 56126 PI Italy

**Keywords:** Trazodone, Astrocytes, Inflammation, Anti-depressant, Neuro-protection

## Abstract

**Background:**

In the central nervous system, glial cells provide metabolic and trophic support to neurons and respond to protracted stress and insults by up-regulating inflammatory processes. Reactive astrocytes and microglia are associated with the pathophysiology of neuronal injury, neurodegenerative diseases and major depression, in both animal models and human brains. Several studies have reported clear anti-inflammatory effects of anti-depressant treatment on astrocytes, especially in models of neurological disorders. Trazodone (TDZ) is a triazolopyridine derivative that is structurally unrelated to other major classes of antidepressants. Although the molecular mechanisms of TDZ in neurons have been investigated, it is unclear whether astrocytes are also a TDZ target.

**Methods:**

The effects of TDZ on human astrocytes were investigated in physiological conditions and following inflammatory insult with lipopolysaccharide (LPS) and tumour necrosis factor-α (TNF-α). Astrocytes were assessed for their responses to pro-inflammatory mediators and cytokines, and the receptors and signalling pathways involved in TDZ-mediated effects were evaluated.

**Results:**

TDZ had no effect on cell proliferation, but it decreased pro-inflammatory mediator release and modulated trophic and transcription factor mRNA expression. Following TDZ treatment, the AKT pathway was activated, whereas extracellular signal-regulated kinase and c-Jun NH_2_-terminal kinase were inhibited. Most importantly, a 72-h TDZ pre-treatment before inflammatory insult completely reversed the anti-proliferative effects induced by LPS-TNF-α. The expression or the activity of inflammatory mediators, including interleukin-6, c-Jun NH_2_-terminal kinase and nuclear factor κB, were also reduced. Furthermore, TDZ affected astrocyte metabolic support to neurons by counteracting the inflammation-mediated lactate decrease. Finally, TDZ protected neuronal-like cells against neurotoxicity mediated by activated astrocytes. These effects mainly involved an activation of 5-HT_1A_ and an antagonism at 5-HT_2A/C_ serotonin receptors. Fluoxetine, used in parallel, showed similar final effects nevertheless it activates different receptors/intracellular pathways.

**Conclusions:**

Altogether, our results demonstrated that TDZ directly acts on astrocytes by regulating intracellular signalling pathways and increasing specific astrocyte-derived neurotrophic factor expression and lactate release. TDZ may contribute to neuronal support by normalizing trophic and metabolic support during neuroinflammation, which is associated with neurological diseases, including major depression.

**Electronic supplementary material:**

The online version of this article (doi:10.1186/s12974-015-0446-x) contains supplementary material, which is available to authorized users.

## Background

Neuroinflammation constitutes an immune response against a diverse spectrum of noxious insults in the central nervous system (CNS), including pathogen invasion, tissue damage, and neurodegenerative processes [1]. Astrocytes are the primary glial cell type in the brain and maintain CNS homeostasis; they promptly respond to injury and regulate neuroinflammatory events [2–4] and are therefore promising targets for modulating neuroinflammation. Both in vitro and in vivo studies have shown that astrocytes produce various cytokines, neurotrophic and growth factors [5–8]. Glial cell over-activation can lead to cytokine-mediated neuronal cell death [9–11], causing neuropathological changes in CNS diseases, such as multiple sclerosis [12], Parkinson’s and Alzheimer’s diseases [13, 14]. Because CNS inflammation is strongly associated with the pathophysiology of depression [15], it is not surprising that astrocyte dysfunction has also been implicated in the neuropathology of major depression. Post-mortem studies of depressed patients revealed reduced numbers and altered morphology of glial cells in cortical regions [16, 17], accompanied by a reduction of astrocytic markers [18, 19]. Furthermore, preclinical studies have shown that glial cell loss in the rat prefrontal cortex is sufficient to induce depressive-like behaviours [20].

Several studies have reported that anti-depressant treatment of glial cells had anti-inflammatory effects, especially under conditions that modelled neurological disorders [21–23]. For example, the commonly used selective serotonin reuptake inhibitor (SSRI) fluoxetine (FLUOX) counteracts astrocytic cell loss in an animal model of depression [24]. Moreover, FLUOX reduces astrocyte glycogen levels and increases glucose utilization and lactate release [25], providing essential energy substrates for neurons to sustain normal function and cellular integrity [26].

Trazodone (TDZ) is a triazolopyridine derivative that is structurally unrelated to other major classes of antidepressants. Its mechanism of action against depression has not been fully elucidated, largely in part to its affinity for several receptors that may contribute to its clinical actions. Unlike SSRIs, TDZ simultaneously inhibits serotonin transporter (SERT), while acting as a partial serotonin 5-HT_1A_ receptor (5-HTR) agonist and a 5-HT_2A_R and 5-HT_2C_R antagonists [27, 28]. Moreover, TDZ exerts antagonistic properties against α_1_- and α_2_-adrenergic receptors (α-AR) and histamine H_1_ receptors with minimal anticholinergic effects [27, 28].

Whereas the molecular and intracellular mechanisms of TDZ have been investigated in neuronal-like cells and animal models [29–31], it is unclear whether astrocytes are a TDZ target. In this study, the effects of TDZ on human astrocytes were investigated by assessing cellular proliferation, cytokine release and trophic and pro-survival gene expression induction. Furthermore, lactate release was investigated to dissect the contribution of TDZ to astrocyte metabolic support of neurons. The intracellular pathways and receptor targets involved in TDZ-elicited effects were investigated; the study was carried out not only under physiological conditions, but also following inflammatory insult with lipopolysaccharide (LPS) and tumour necrosis factor-α (TNF-α) [32].

## Methods

### Materials

Human astrocytes were purchased from GIBCO (Life Technologies, Milan, Italy). ELISA kits for cytokines’ determination were from Thermo Fisher Scientific, Rodano, Milan, Italy. All other reagents were obtained from standard commercial sources and were of the highest commercially available grade.

### Cell culture, pharmacological treatments and induction of inflammation

Human astrocytes were cultured in complete medium consisting of DMEM, 1 % N-2 Supplement (Life Technologies, Milan, Italy), 10 % fetal bovine serum (FBS), and 2 mM L-glutamine, at 37 °C in 5 % CO_2_. H9-derived human neural stem cells (NSCs) were purchased from GIBCO (Life Technologies, Milan, Italy). For neuronal differentiation, H9-derived NSCs were plated on polyornithine and laminin-coated culture dishes, and switched into a defined Neurobasal serum-free medium, containing 2 % B-27, 2 mM L-glutamine and 5 μM retinoic acid (RA) up to 7 days, as reported before [29].

To set up the inflammation model, human astrocytes were incubated for 2, 6 or 24 h with LPS (50 μg/ml) and/or TNF-α (50 ng/ml), commonly used as inflammation inductors [33–35].

### Cell proliferation assays

Trazodone (TDZ, Angelini Acraf S.p.a.) was diluted to different concentrations of stock solutions by saline solution. After replating, human astrocytes (5000 cells/well) were treated once with different concentrations of TDZ (1 nM-10 μM), 10 μM fluoxetine (FLUOX) or 10 μM serotonin (5-HT) for 24 or 72 h. To verify the protective effects of the drugs, human astrocytes were treated with TDZ, FLUOX or 5-HT for 24 or 72 h; following incubation time, cells were washed and incubated with LPS-TNF-α for an additional 24 h. In longer treatments (7 days, Additional file 1: Figure S1), drug treatment was repeated every three days.

To dissect the receptor targets involved in TDZ- and FLUOX-mediated effects, human astrocytes were pre-treated for 30 min with the following selective receptor activators/blockers: 250 nM clonidine (α-adrenergic agonist, [36]), or 100 μM histamine, or 15 nM (S)-WAY 100135 dihydrochloride (selective 5-HT_1A_R antagonist [37]), 10 nM GR 127935 hydrochloride (5-HT_1B/D_R antagonist [38]), or 5 nM RS 127445 hydrochloride (5-HT_2B_R antagonist [39]), 30 nM (R)-1-(2,5-dimethoxy-4-iodophenyl)-2-aminopropane [(R)-DOI], (5-HT_2A/C_R agonist, [40]), or 100 nM SR 57227 (5-HT_3_R agonist, [41]). After pre-incubation, cells were treated with TDZ (1 nM-10 μM) or 10 μM FLUOX for 72 h, followed by LPS-TNF-α for an additional 24 h.

At the end of treatments, cell proliferation was determined using the MTS assay according to manufacturer’s instruction. Within an experiment, each condition was assayed in triplicate, and each experiment was performed at least three times. The results were calculated by subtracting the mean background from the values obtained from each test condition and were expressed as the percentage of the control (untreated cells).

### RNA extraction and real-time PCR analysis

Human astrocytes were treated with medium alone (control), TDZ (100 nM or 1 μM), FLUOX (10 μM) for 24 or 72 h. When indicated, after drug removal, cells were incubated with LPS-TNF-α for additional 24 h. When indicated, cells were pre-incubated with 500 nM Wortmannin (Phosphatidylinositol-4,5-bisphosphate 3-kinase, PI3K, inhibitor) or 5 μM PD98059 (a highly selective in vitro MEK1 inhibitor, [42]) for 30 min, before astrocyte treatment with 100 nM TDZ.

At the end of treatments, cells were collected, and total RNA was extracted using Rneasy® Mini Kit (Qiagen, Hilden, Germany) according to manufacturer’s instructions. Purity of the RNA samples was determined by measuring the absorbance at 260:280 nm. cDNA synthesis was performed with 500 ng of RNA using i-Script cDNA synthesis kit (BioRad, Hercules, USA). Primers used for RT-PCR were designed in intron/exon boundaries to ensure that products did not include genomic DNA [29]. RT-PCR reactions consisted of 25 μL Fluocycle® II SYBR® (Euroclone, Milan, Italy), 1.5 μL of both 10 μM forward and reverse primers, 3 μL cDNA, and 19 μL of H_2_O. All reactions were performed for 40 cycles using the following temperature profiles: 98 °C for 30 s (initial denaturation); T °C (see Table 1) for 30 s (annealing); and 72 °C for 3 s (extension) [29]. β-actin was used as the housekeeping gene. PCR specificity was determined by both the melting curve analysis and gel electrophoresis, and the data was analysed by standard curve method.

**Table 1:** Nucleotide sequences, annealing temperature and product size of the primers utilised in PCR experiments

Gene	Primer nucleotide sequences	Product size (base pairs)	Annealing temperature
CREB	FOR: 5’-AAGCTGAAAGTCAACAAATGACA-3’	240	52 °C
	REV: 5’-CCTCTTTTCAGAAAAATTCAGGA-3’		
BDNF	FOR: 5’-TACATTTGTATGTTGTGAAGATGTTT-3’	131	56 °C
	REV: 5’- TTACTCGCCCCGGACCCTCT-3’		
NF-kB	FOR: 5’- GCTCCGGAGACCCCTTCCA-3’	198	54 °C
	REV: 5’- GGTTTGAGGTAGTTTCCCAGT-3’		
mTOR	FOR: 5’-CCGTTCCATCTCCTTGTCACG-3’	209	56 °C
	REV: 5’-CCACTTACTCTGCAGTGTG-3’		
β-actin	FOR: 5’-GCACTCTTCCAGCCTTCCTTCC-3’	254	55 °C
	REV-5’-GAGCCGCCGATCCACACG-3’		

### Cytokine and lactate release

Human astrocytes were treated with medium alone (control), TDZ (1 nM-10 μM), FLUOX (10 μM), or 5-HT (10 μM) for 24 or 72 h. When indicated, after drug removal, cells were incubated with LPS and TNF-α for an additional 24 h. The amount of cytokines (i.e., interleukin*-*6, IL-6, IL-10 and interferon gamma, IFN-γ) or lactate released into the culture medium was measured using ELISA kits (Thermo Fisher Scientific, Rodano, Milan, Italy, and Sigma Aldrich, Milan, Italy) following the manufacturers’ instructions. Culture supernatants were collected and stored at −80 °C until assayed for cytokine or lactate content.

### Astrocyte conditioned media

Human H9 NSCs were differentiated to neuronal-like cells using a defined Neurobasal serum-free medium, containing 2 % B-27, 2 mM L-glutamine and 5 μM retinoic acid up to 7 days. Neuronal-like cells were plated in 96-well plates at a density of 4000 cells per well and allowed to settle for 24 h at 37 °C before replacement with conditioned media. Human astrocytes were treated with medium alone (control), TDZ (1 nM-10 μM), FLUOX (10 μM) or 5-HT (10 μM) for 24 or 72 h. In some experiments, after TDZ, FLUOX and 5-HT removal, cells were incubated with LPS and TNF-α for an additional 24 h. Culture media were collected as conditioned media and clarified by centrifugation at 20,000×*g* for 5 min to remove cellular debris. The media were then transferred onto neuronal-like cells, whose prolifer+ation was measured using the MTS assay as described above after 24 h of incubation (see Fig. 8a).

### MAPK (mitogen-activated phosphorylation kinase) assays

Astrocytes were treated with medium alone (control), TDZ (1 nM-10 μM), or FLUOX (10 μM) or 5-HT (10 μM), for 30 min or 24 h. In some experiments, before incubation with TDZ (10 μM) or FLUOX (10 μM), cells were pre-treated for 15 min with the following selective receptor activators/inhibitors: 250 nM clonidine (α-adrenergic agonist), or 100 μM histamine, or 15 nM (S)-WAY 100135 dihydrochloride (selective 5-HTR_1A_R antagonist), 10 nM GR 127935 hydrochloride (5-HTR_1B/D_R antagonist), or 5 nM RS 127445 hydrochloride (5-HT_2B_R antagonist) or 30 nM (*R*)-DOI (5-HT_2A/C_R agonist), or 100 nM SR 57227 (5-HT_3_R agonist). Similarly, human astrocytes were pre-treated with 1 μM H89 (Protein Kinase A, PKA inhibitor), or 1 μM bisindolylmaleimide (PKC inhibitor), or 500 nM wortmannin (PI3K, inhibitor). Finally, to block G_αi/o_ proteins, cells were pre-incubated in non-complete medium with 200 ng/ml pertussin toxin (PTX) for 18 h.

When indicated, after TDZ or FLUOX treatment for 24 or 72 h, the drugs were removed, and astrocytes were incubated with LPS and TNF-α for additional 24 h.

At the end of treatments, cells were fixed with 4 % formaldehyde to preserve activation of specific protein modification. Levels of total and phosphorylated AKT, c-Jun N-terminal kinases (JNKs) and extracellular signal-regulated kinases (ERK1/2) were determined by ELISA assays, as previously reported [29, 43]. Briefly, the cells were washed three times with wash buffer (0.1 % Triton X-100 in PBS) and 100 μl of quenching buffer (1 % H_2_O_2_; 0.1 % sodium azide in wash buffer) was added and incubation was protracted for other 20 min. The cells were washed with PBS twice, and then 100 μl of blocking solution (1 % BSA; 0.1 % Triton X-100 in PBS) was added for 60 min. After blocking, cells were washed three times with wash buffer and the specific primary antibodies (anti-phospho ERK1/2, 1:500, sc-7383 Santa Cruz Biotechnology; anti-ERK1/2, 1:500, #4695 Cell Signaling Technology; anti-phospho JNK, 1:500, sc-6254 SantaCruz Biotechnology; anti-JNK, SAB4200176, 1:750, Sigma Aldrich, Milan, Italy; anti-phospho AKT, 1:500, sc-7985 Santa Cruz Biotechnology; anti-AKT, 1:1000, SAB4500799, Sigma Aldrich, Milan, Italy) were added on at 4 °C. Subsequent incubation with secondary HRP-conjugated antibodies and developing solution allowed a colorimetric quantification of total and phosphorylated levels. Blanks were obtained processing wells without cells in the absence of the primary antibody. The relative number of cells in each well was then determined using Crystal Violet solution. The results were calculated by subtracting the mean background from the values obtained from each test condition; values were normalized to the number of cells in each well, and were expressed as the percentage of untreated cells (basal).

### ERK overexpression

Cells were transfected with human CMV6-XL4 MAPK (Origene, Rockville, MD, USA) or with the plasmid alone by the PEI method (JetPEI, Polyplus transfection, [44]). Two days after transfection, astrocytes were trypsinized and seeded in medium containing 200 μg/ml ampicillin. MAPK overexpression was verified by western blot, as described below.

### Western blot analysis

Astrocytes were treated with medium alone (control), TDZ (1 or 10 μM), or FLUOX (10 μM) or Rapamycin (RAPA, 15 nM) for 24 or 72 h. At the end of the treatment period, the cells were collected and then were lysed for 60 min at 4 °C using 200 μL of RIPA buffer (9.1 mM NaH_2_PO_4_, 1.7 mM Na_2_HPO_4_, 150 mM NaCl, pH 7.4, 0.5 % sodium deoxycholate, 1 % Nonidet P-40, 0.1 % SDS, and a protease-inhibitor cocktail). Equal amounts of the cell extracts (40 μg of protein) were diluted in Laemmli sample solution, resolved using SDS-PAGE (8.5 %), transferred to PVDF membranes and probed overnight at 4 °C using the following primary antibodies: anti-phospho ERK1/2, 1:500, sc-7383 Santa Cruz Biotechnology; anti-ERK1/2, 1:1000, #4695 Cell Signaling Technology; anti-phospho JNK, 1:500, sc-6254 SantaCruz Biotechnology; anti-JNK, SAB4200176, 1:750, Sigma Aldrich, Milan, Italy; anti-phospho AKT, 1:500, sc-7985 Santa Cruz Biotechnology; anti-AKT, 1:1000, SAB4500799, Sigma Aldrich, Milan, Italy; anti-microtubule-associated protein light chain-3 (LC3B, sc-28266 Santa Cruz Biotechnology, 1:100); anti-glial fibrillary acidic protein, GFAP (sc-9065, Santa Cruz Biotechnology 1:50) and glyceraldehyde-3-phosphate dehydrogenase, GAPDH (G9545, Sigma Aldrich, Milan, Italy, 1:5000). The primary antibodies were detected using the appropriate peroxidase-conjugated secondary antibodies, which were then detected using a chemiluminescent substrate (ECL, Perkin Elmer). Densitometric analysis of the immunoreactive bands was performed using Image J Software.

### NF-κB activation

Human astrocytes were treated with medium alone (control) or TDZ (1 μM) for 24 or 72 h; after drug removal, cells were incubated with LPS-TNF-α for an 24 h. At the end of treatment, cells were lysed in the “subcellular fractionation buffer” (250 mM Sucrose, 20 mM HEPES, 10 mM KCl, 1.5 mM MgCl_2_, 1 mM EDTA, 1 mM EGTA, pH 7.4), containing 1 mM DTT and protease-inhibitor cocktail. The cells were centrifuged at 1,000 x g for 10 min to isolate nuclei, and the supernatants were centrifuged again at 10,000×*g* to obtain the cytosolic/membrane fraction. Nuclei were suspended in the subcellular fractionation buffer; the protein levels of NF-kB p65 were evaluated in cytoplasmic and nuclear extracts (40 μg) by western blot analysis, as described before, using the following primary antibodies: anti-NF-κB p65, 1:300, sc-372 Santa Cruz Biotechnology; histone H3 (nuclear marker), 1:500, sc-10809 Santa Cruz Biotechnology; GAPDH (cytoplasmatic marker).

### CREB activation

Human astrocytes were treated with medium alone (control), TDZ (1 μM), FLUOX (10 μM) for 24 or 72 h. After drug removal, cells were incubated with LPS-TNF-α for additional 24 h. Levels of phosphorylated and total CREB were detected by the high-throughput TransAM® assay (Active Motif, La Hulpe, Belgium), following the manufacturer’s instructions.

### Statistical analysis

The nonlinear multipurpose curve-fitting program Graph-Pad Prism (GraphPad Software Inc., San Diego, CA) was used for data analysis and graphic presentations. All data are presented as the mean ± SEM. Statistical analysis was performed by one-way analysis of variance (ANOVA) with Tukey HSD corrected *t* test for post hoc pair-wise comparisons. *P* < 0.05 was considered statistically significant.

## Results

### Effect of TDZ treatment on astrocyte proliferation

**Fig. 1:**
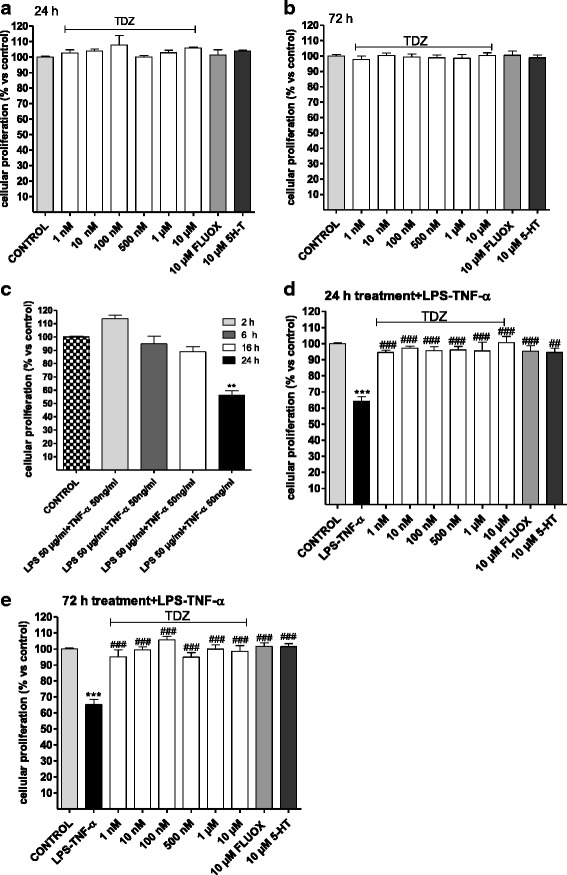
Effect of TDZ on astrocyte proliferation and on an experimental inflammation model. **a**, **b** Human astrocytes were treated with medium alone (control), or different concentrations of TDZ (1 nM-10 μM), or FLUOX (10 μM) or 5-HT (10 μM) for 24 h (**a**) or 72 h (**b**). At the end of the treatments, cell proliferation was measured by MTS assay. The data are expressed as percentages relative to untreated cells (control), which were set at 100 %, and represent the mean ± SEM of three independent experiments, each performed in triplicate. Statistical significance was determined using a one-way ANOVA-Tukey HSD post hoc test. **c** Human astrocytes were treated with 50 μg/ml LPS and 50 ng/ml TNF-α for different time periods (2–24 h). **d**, **e** Human astrocytes were treated with different concentrations of TDZ (1 nM-10 μM), or FLUOX (10 μM) or 5-HT (10 μM) for 24 h (**d**) or 72 h (**e**); after drug removal, cells were incubated with 50 μg/ml LPS and 50 ng/ml TNF-α for an additional 24 h. At the end of treatment, cell proliferation was measured by MTS assay. The data are expressed as percentages relative to untreated cells (control), which were set at 100 %, and represent the mean ± SEM of three independent experiments, each performed in triplicate. Statistical significance was determined using a one-way ANOVA-Tukey HSD post hoc test: **P < 0.001, ***P < 0.001 vs. control; ^##^ *P* < 0.01, ^###^ *P* < 0.001 vs. cells treated with LPS-TNF-α

To verify whether TDZ treatment affected astrocyte proliferation, human cells were treated with different concentrations of the drug (1 nM-10 μM) for 24 or 72 h. As shown in Fig. 1a, b, 24 and 72 h of TDZ incubation did not induce any significant changes in astrocyte proliferation at any concentration tested. Similar results were obtained with FLUOX or 5-HT (Fig. 1a, b).

The effects of TDZ treatment on glial inflammation were then investigated. To activate astrocytes, cells were treated with 50 μg/ml LPS and 50 ng/ml TNF-α for various time periods. Whereas no significant reduction in astrocyte proliferation was observed within 16 h of inflammatory insult (Fig. 1c), LPS-TNF-α challenge for 24 h decreased the percentage of viable/proliferative astrocytes to 43.7 ± 3.3 % (Fig. 1c). Therefore, the latter condition was chosen for the experimental model of in vitro astrocyte activation*.*

Upon 24 h TDZ, FLUOX or 5-HT pre-treatment before inflammatory insult induction, a significant increase in proliferation at all concentrations examined was observed (Fig. 1d). Similar results were obtained when cells were pre-treated for 72 h (Fig. 1e). These results suggest that TDZ exerts cyto-protective effects on human astrocytes in an experimental model of inflammation.

### Effect of TDZ treatment on cytokine release

To evaluate the effect of TDZ on astrocyte cytokine production, pro- (i.e. IL-6 and IFN-γ) and anti-inflammatory (i.e. IL-10) cytokine release were analysed. Astrocytes were challenged with LPS-TNF-α for 24 h in the presence or absence of drug pre-treatment.

**Fig. 2:**
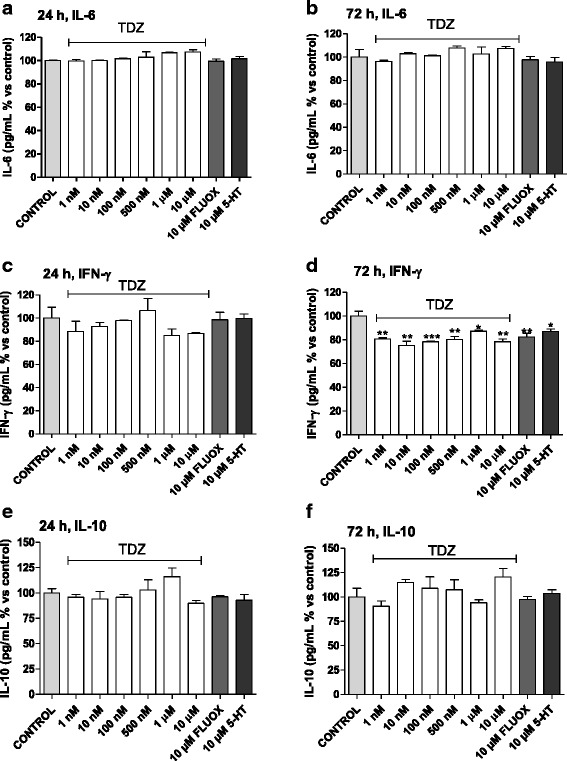
Effect of TDZ on cytokine release. Human astrocytes were treated with different concentrations of TDZ (1 nM-10 μM), or FLUOX (10 μM) or 5-HT (10 μM) for 24 h (**a**, **c**, **e**) or 72 h (**b**, **d**, **f**). At the end of treatment, culture supernatants were collected, and the amounts of IL-6 (**a**, **b**), IFN-γ (**c**, **d**) and IL-10 (**e, f**) released were measured using ELISA kits following the manufacturer’s instructions. The data are expressed as percentages relative to untreated cells (control), which were set at 100 %, and represent the mean ± SEM of two independent experiments, each performed in duplicate. Statistical significance was determined using a one-way ANOVA-Tukey HSD post hoc test: *P < 0.05, **P < 0.01, ***P < 0.001 vs. control

At all concentrations tested, TDZ, FLUOX or 5-HT alone did not markedly alter IL-6, IFN-γ or IL-10 release after 24 h of treatment (Fig. 2a, c, e). In contrast, 72-h TDZ treatment significantly reduced IFN-γ release, without altering IL-6 or IL-10 production (Fig. 2b, d, f). Similar results were obtained upon cell treatment with FLUOX and 5-HT (Fig. 2b, d, f).

**Fig. 3:**
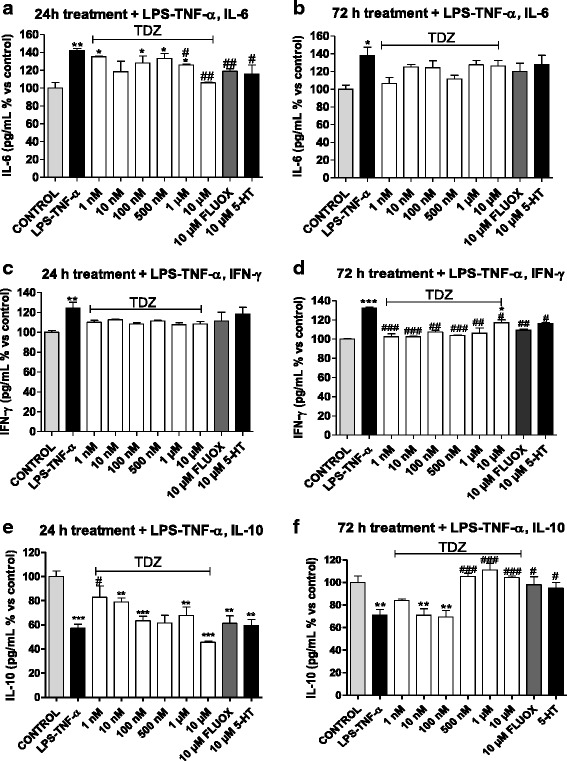
Cytokine release in an experimental model of inflammation. Human astrocytes were treated with medium alone (control), or different concentrations of TDZ (1 nM-10 μM), or FLUOX (10 μM) or 5-HT (10 μM) for 24 h (**a**, **c**, **e**) or 72 h (**b**, **d**, **f**); after drug removal, cells were incubated with 50 μg/ml LPS and 50 ng/ml TNF-α for an additional 24 h. At the end of treatments, supernatants were collected, and the amounts of IL-6 (**a, b**), IFN-γ (**c, d**) and IL-10 (**e, f**) were measured using ELISA kits following the manufacturer’s instructions. The data are expressed as percentages relative to untreated cells (control), which were set at 100 %, and represent the mean ± SEM of two independent experiments, each performed in duplicate. Statistical significance was determined using a one-way ANOVA-Tukey HSD post hoc test: *P < 0.05, **P < 0.01, ***P < 0.001 vs. control; ^#^ *P* < 0.05, ^##^ *P* < 0.01, ^###^ *P* < 0.001 vs. LPS-TNF-α-treated cells

As expected, inflammatory insult (LPS-TNF-α) significantly elevated IL-6 and IFN-γ levels and reduced basal IL-10 release (Fig. 3a–f), consistent with previous reports [7, 45].

Astrocyte challenge with micromolar concentrations of TDZ for 24 h before inflammatory damage significantly prevented IL-6 release (Fig. 3a). Moreover, a trend toward IFN-γ reduction (Fig. 3c) and IL-10 increase (Fig. 3e) was evidenced.

After 72 h pre-treatment, IFN-γ release was significantly prevented at all concentrations tested (Fig. 3d). Moreover, TDZ counteracted the LPS-TNF-α-induced decrease in IL-10 levels at a range of concentrations between 500 nM and 10 μM (Fig. 3f).

In a similar manner, FLUOX and 5-HT prevented IL-6 release after 24 h (Fig. 3a), while they reduced IFN-γ release (Fig. 3d) and counteracted IL-10 reduction (Fig. 3f) in astrocytes pre-incubated for 72 h. These data may suggest that IL-6 is involved in the 24-h pre-treatment before induction of inflammation, while IFN-γ and IL-10 can play major roles in a longer pre-treatment with anti-depressant drugs.

Taken together, these results demonstrate that TDZ pre-treatment of human astrocytes, particularly for 72 h, reduced pro-inflammatory cytokine release and counteracted the reduction in anti-inflammatory cytokine release.

### Effect of TDZ treatment on pro-inflammatory gene, trophic factor and transcription factor expression

Nuclear factor kappa B (NF-κB) is a crucial regulator of immunity and inflammation and has been shown to be pivotal for astrocytic neuroinflammatory responses [46]. Therefore, the effects of TDZ treatment on NF-kB expression were analysed in the absence and presence of astrocyte activation with LPS-TNF-α. In parallel, the expression of trophic and transcription factors was investigated.

**Fig. 4:**
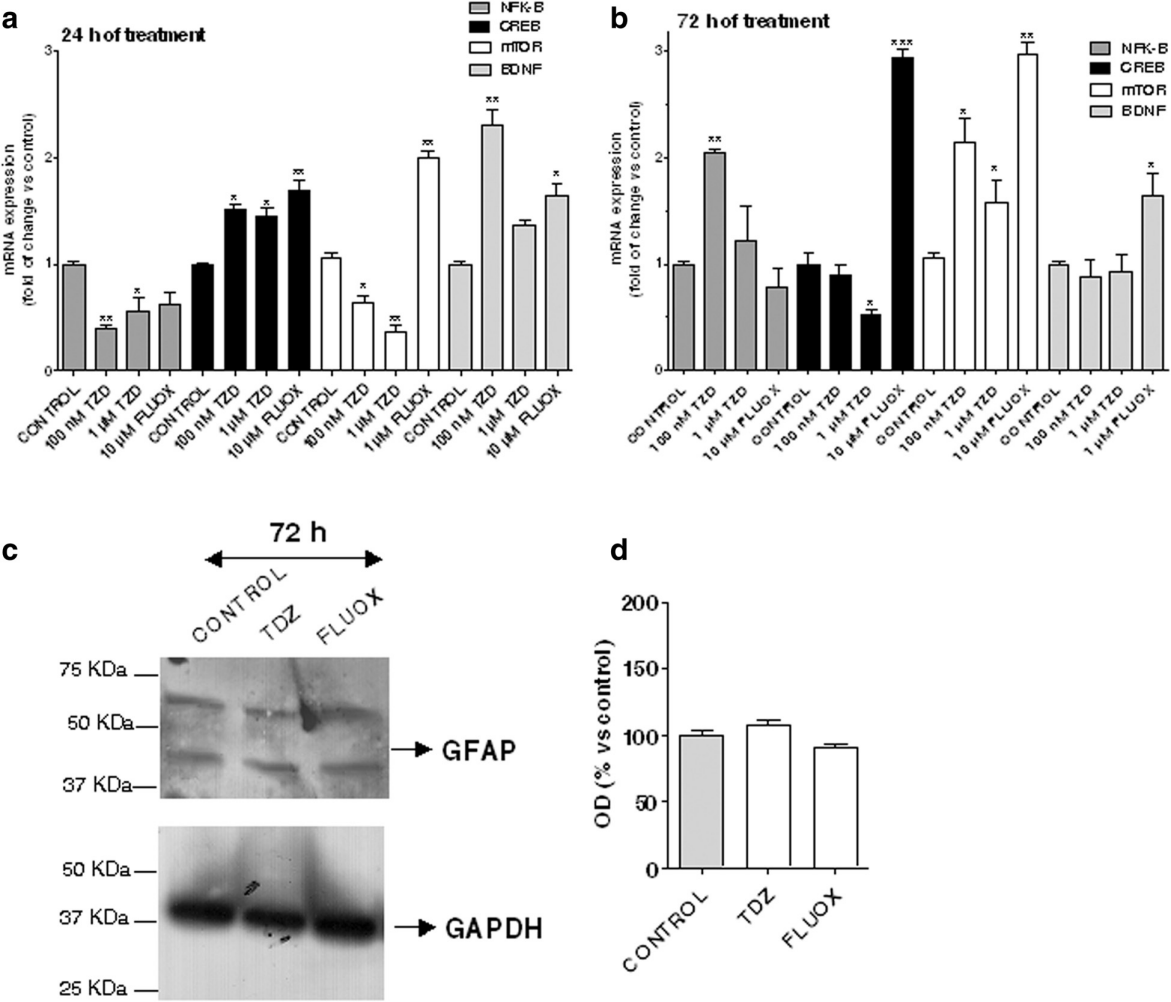
Effect of TDZ on pro-inflammatory gene, trophic and transcription factor expression. **a**, **b** Human astrocytes were treated with medium alone (control), or different concentrations of TDZ (100 nM-1 μM) or FLUOX (10 μM) for 24 h (**a**) or 72 h (**b**). At the end of treatment, total RNA was extracted, and relative mRNA quantification of NF-kB, CREB, mTOR and BDNF was performed by real-time RT-PCR*.* The data are expressed as fold changes vs. control and represent the mean ± SEM of three different experiments, each performed in duplicate. Statistical significance was determined using a one-way ANOVA-Tukey HSD post hoc test: *P < 0.05, **P < 0.01, ***P < 0.001 vs. control. **c**, **d** Human astrocytes were treated with medium alone (control), TDZ (10 μM) or FLUOX (10 μM) for 72 h. Following incubation, the protein levels of GFAP were evaluated by western blot analysis. GAPDH was the loading control. **c** Representative western blots. **d** Densitometric analysis of the immunoreactive bands was performed using ImageJ. The data are expressed as the percentage of optical density of the immunoreactive band relative to that of the control, which was set at 100 %, and are the mean values ± SEM of three different experiments. Statistical significance was determined using a one-way ANOVA-Tukey HSD post hoc test

After 24 h of TDZ treatment, a significant up-regulation of the mRNA levels of brain-derived nerve factor (BDNF) and the cAMP response element-binding protein (CREB) was observed (Fig. 4a), suggesting that TDZ alone induced the expression trophic and transcription factors. In contrast, the pro-inflammatory gene NF-kB and mammalian target of rapamycin (mTOR) mRNA levels significantly decreased (Fig. 4a). These results confirmed the anti-inflammatory effect of TDZ, as mTOR inhibition has been demonstrated to elicit anti-inflammatory effects in glial cells [47].

As a comparison, astrocyte incubation with FLUOX showed similar results both after 24 or 72 h of cell treatment: the SSRI enhanced CREB, BDNF and mTOR mRNA levels, without significantly altering NF-kB transcription (Fig. 4a, b). These data are consistent with previous reports on the SSRI drug [25]. Surprisingly, a 72-h TDZ challenge had opposite effects; it significantly increased NF-kB and mTOR mRNA levels and reduced CREB transcription (Fig. 4b).

To dissect if a long term treatment with the anti-depressant could activate astrocytes, the levels of the astrocyte-specific activation marker GFAP were measured. Western blot analysis did not show any significant increase of GFAP in TDZ-treated samples (Fig. 4c, d). Consistent with these data, astrocyte proliferation was not affected even after 7 days of TDZ treatment (Additional file 1: Figure S1).

**Fig. 5:**
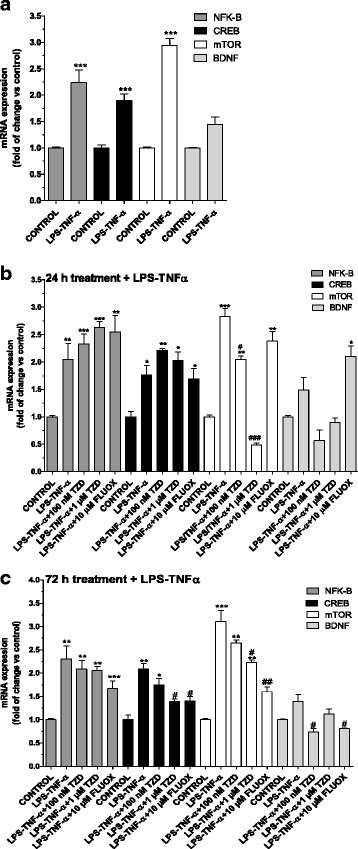
Expression of pro-inflammatory genes, trophic and transcription factors in an experimental model of inflammation. **a** Human astrocytes were treated with 50 μg/ml LPS and 50 ng/ml TNF-α for 24 h. **b**, **c** Human astrocytes were treated with medium alone (control), different concentrations of TDZ (100 nM-1 μM), or FLUOX (10 μM) for 24 h (**b**) or 72 h (**c**); after drug removal, cells were incubated with 50 μg/ml LPS and 50 ng/ml TNF-α for an additional 24 h. At the end of treatment, total RNA was extracted, and relative mRNA quantification of NF-kB, CREB, mTOR and BDNF was performed by RT-PCR*.* The data are expressed as fold changes vs. control and represent the mean ± SEM of three different experiments, each performed in duplicate. Statistical significance was determined using a one-way ANOVA-Tukey HSD post hoc test: *P < 0.05, **P < 0.01, ***P < 0.001 vs. control; ^#^ *P* < 0.05, ^##*P*^ < 0.01, ^###^ *P* < 0.001 vs. cells treated with LPS-TNF-α

The effects of the inflammatory damage on the expression of trophic and transcription factors were then determined. LPS-TNF-α treatment significantly increased NF-kB levels (Fig. 5a), consistent with its role as a pro-inflammatory gene [46]. Moreover, mTOR and CREB levels were significantly increased (Fig. 5a). These results are consistent with previous data from LPS or TNF-α-stimulated glial cells [48, 49]. Pre-incubation of astrocytes with TDZ or FLUOX for 24 h further increased inflammation-mediated increase in NF-kB mRNA expression, suggesting that 24-h pre-treatment with antidepressants is not sufficient to reverse the effects of LPS-TNF-α on NF-kB induction. In contrast, a significant decrease in mTOR levels after 24-h TDZ pre-incubation was evidenced (Fig. 5b).

TDZ treatment for 72 h significantly counteracted the LPS-TNF-α-induced increase in NF-kB, CREB and mTOR expression (Fig. 5c). Α decrease in BDNF expression was also observed. Similar results were observed upon FLUOX treatment (Fig. 5c). These data demonstrate that TDZ pre-incubation for 72 h counteracts the induction of the pro-inflammatory genes mediated by LPS-TNF-α.

**Fig. 6:**
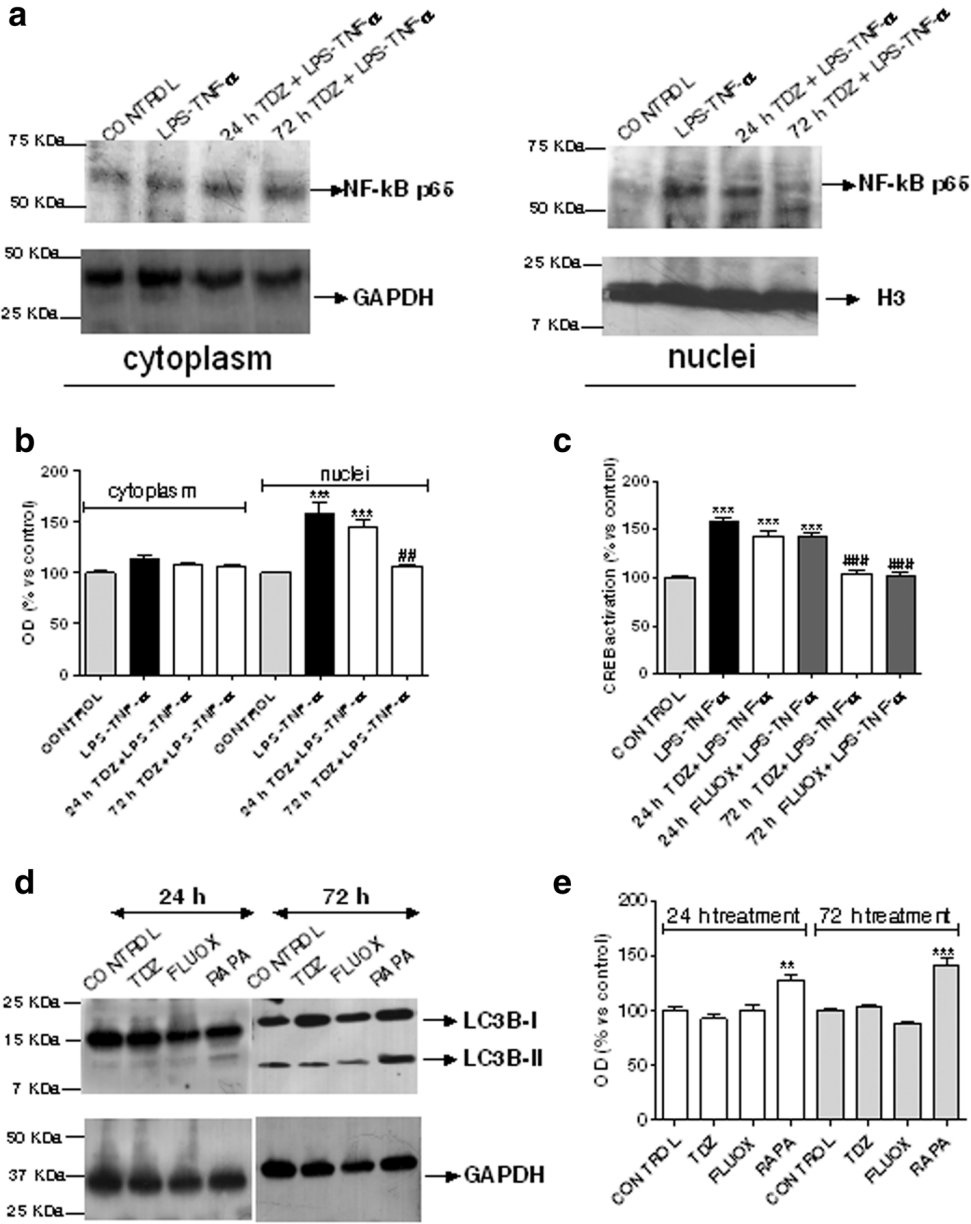
Effect of TDZ on NF-kB /CREB activation and on the autophagic pathway. **a**, **b** Human astrocytes were treated with medium alone or TDZ (1 μM) for 24 or 72 h, and then with LPS-TNF-α for an additional 24 h. NF-kB p65 protein levels were evaluated in cytoplasm and nuclei by western blot analysis. GAPDH and H3 were the loading controls. **a** Representative western blots. **b** Densitometric analysis of the immunoreactive bands was performed using ImageJ. The data are expressed as the percentage of optical density of the immunoreactive band relative to that of the control, which was set at 100 % and are the mean values ± SEM of three different experiments. **c** Human astrocytes were treated with medium alone, or TDZ (1 μM) or FLUOX (10 μM) for 24 h or 72 h, and then with LPS-TNF-α for an additional 24 h. At the end of treatment, CREB activation was determined by ELISA, as described in the Methods section. The data are expressed as percentages relative to untreated cells (control), which were set at 100 %, and represent the mean ± SEM of two independent experiments, each performed in duplicate. **d**, **e **Human astrocytes were treated with medium alone, TDZ or FLUOX or Rapamycin (RAPA) for 24 or 72 h. Following incubation, the protein levels of LC3B were evaluated by western blot analysis. GAPDH was the loading control. **d** Representative western blots. **e** Densitometric analysis of the immunoreactive bands. The data are expressed as the percentage of optical density of the immunoreactive band relative to that of the control, which was set at 100 %, and are the mean values ± SEM of three different experiments. Statistical significance was determined using a one-way ANOVA-Tukey HSD post hoc test: **P < 0.01, ***P < 0.001 vs. control; ^##^ *P* < 0.01, ^###^ *P* < 0.001 vs. cells treated with LPS-TNF-α

Following stimuli that elicit the NF-kB pathway, its transcription factor subunits, p65 and p50, expose nuclear targeting signals and then translocate into the nucleus [50]. Consistent with real-time PCR data, LPS-TNF-α induced a significant p65 nuclear accumulation (Fig. 6a, b); astrocyte pre-treatment for 72 h with TDZ (1 μM) significantly counteracted LPS-TNF-α-mediated NF-kB activation (Fig. 6a, b).

ELISA experiments showed that the inflammatory stimuli caused CREB phosphorylation (Fig. 6c), consistent with literature [49] and real-time PCR data (Fig. 5a). Astrocyte pre-treatment for 72 h with TDZ or FLUOX significantly decreased CREB activation induced by LPS-TNF-α (Fig. 6c). Globally, these data confirmed that TDZ pre-incubation for 72 h counteracts the induction of the pro-inflammatory mediators elicited by LPS-TNF-α.

### Effect of TDZ treatment on the autophagic pathway

Given recent publications on the link between antidepressants and autophagy [51, 52], and the modulation of mTOR mRNA in TDZ-treated samples, the effects of TDZ on astrocytic autophagy was verified. During the autophagic process, the microtubule-associated protein light chain-3 (LC3B-I) becomes conjugated with phosphatidyl ethanolamine to form LC3B-II that integrates into the autophagosomal membrane [53]. In astrocytes treated with TDZ or FLUOX for 24 or 72 h, any significant LC3B-I-to-LC3B-II conversion indicative of autophagosome formation was evidenced (Fig. 6d, e), as compared with the standard mTOR inhibitor rapamycin [54]. These data demonstrate that neither TDZ nor FLUOX affect autophagy pathways in human astrocytes.

Consistent with our data, selective SSRIs have been demonstrated to not induce autophagy [52], while in a different paper, the SSRI paroxetine has been reported to cause cell autophagy in mice and human cells [55].

### Effects of TDZ treatments on lactate release

In addition to supplying neurotrophic/growth factors, astrocytes provide essential energy substrates to neurons. In particular, astrocytes take up glucose and glycolytically metabolize it to lactate, which is then released in the extracellular space and oxidized by neurons to meet part of their energy needs [56]. These observations led us to examine whether TDZ alters lactate release from human astrocytes.

**Fig. 7:**
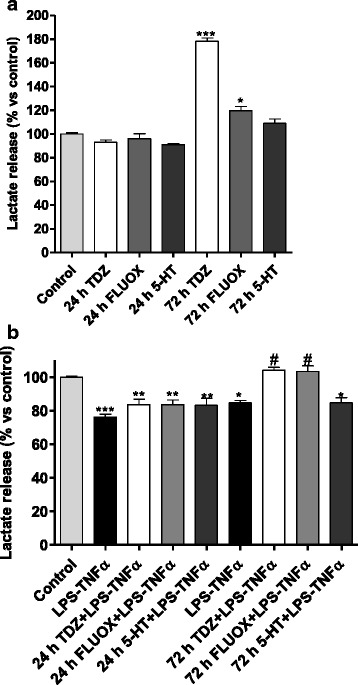
Effect of TDZ on lactate release. **a** Human astrocytes were treated with medium alone (control), or TDZ (10 μM), or FLUOX (10 μM) or 5-HT (10 μM) for 24 or 72 h. **b** Cells were treated as in **a**. After drug removal, cells were incubated with LPS-TNF-α for additional 24 h. At the end of treatment, supernatants were collected, and the amounts of lactate released were measured using an ELISA kit according to the manufacturer’s instructions. The data are expressed as percentages relative to untreated cells (control), which were set at 100 %, and represent the mean ± SEM of two independent experiments, each performed in duplicate. Statistical significance was determined using a one-way ANOVA followed by a Bonferroni post-test: *P < 0.05, **P < 0.01, ***P < 0.001 vs. control; ^#^ *P* < 0.05 vs. cells treated with LPS-TNF-α

As shown in Fig. 7a, TDZ treatment for 72 h significantly increased lactate release; the same was not observed for the 24-h treatment. Similar results were obtained in the presence of FLUOX, but not of the endogenous transmitter 5-HT (Fig. 7a).

Interestingly, LPS-TNF-α significantly decreased the amount of lactate in the culture media (Fig. 7b). TDZ or FLUOX treatment for 24 h before inflammation induction did not affect the inflammation-induced decrease in lactate (Fig. 7b), confirming that a 24-h incubation with anti-depressant is not sufficient to induce significant changes in astrocyte metabolism. In contrast, drug pre-treatment for 72 h completely reversed the LPS-TNF-α-induced decrease in lactate release (Fig. 7b), suggesting that TDZ protective effects may involve changes in cell metabolism too.

### Effects of TDZ on astrocyte-mediated neurotoxicity

**Fig. 8:**
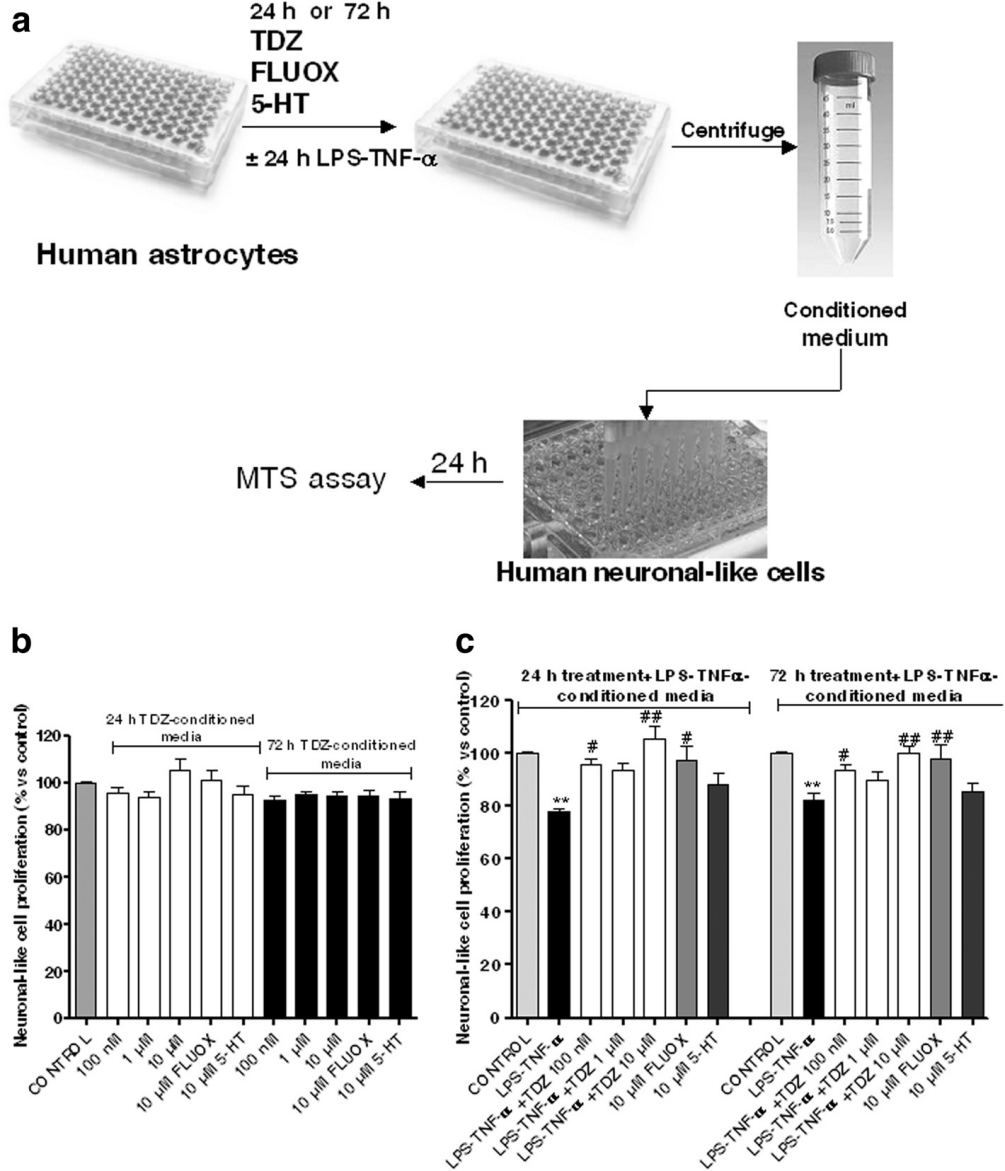
Effects of TDZ on astrocyte-mediated neurotoxicity. **a**, **b**, **c** Human astrocytes were first treated with the indicated concentrations of TDZ, FLUOX or 5-HT for 24 or 72 h (**b**); after TDZ removal, cells were incubated with LPS-TNF-α for an additional 24 h (**c**). At the end of treatment, the media were collected as conditioned media and added to neuronal-like cells for 24 h. **a** Scheme of treatment. **b**, **c** Neuronal-like cell proliferation was measured by MTS assay. The data are expressed as percentages relative to untreated cells (control), which were set at 100 %, and represent the mean ± SEM of three independent experiments, each performed in triplicate. Statistical significance was determined using a one-way ANOVA followed by a Bonferroni post-test: **P < 0.01 vs. control; ^#^ *P* < 0.05, ^##^ *P* < 0.01 vs. cells treated with LPS-TNF-α

Reactive astrocytes can induce neuronal cell degeneration by releasing inflammatory mediators and cytokines [57]. We, therefore, investigated whether TDZ contributes to the relief of activated astrocyte-induced neurotoxicity (Fig. 8a). As shown in Fig. 8b, conditioned media from 24 or 72 h 5-HT-, FLUOX- or TDZ-treated astrocytes did not significantly affect neuronal-like cell proliferation. In contrast, conditioned media from LPS-TNF-α-stimulated astrocytes significantly decreased neuronal-like cell proliferation (Fig. 8c). Upon 24 or 72 h TDZ pre-treatment prior to LPS-TNF-α stimulation (Fig. 8c), LPS-TNF-α-induced effects were significantly counteracted. Similar results were obtained with FLUOX, and in a minor extent, with 5-HT (Fig. 8c), suggesting that the two antidepressants suppress neurotoxicity mediated by activated astrocytes.

### Contributions of the 5-HT receptors to TDZ pro-survival effects

To dissect the putative contribution of 5-HT receptors to the protective effects elicited by TDZ, viability experiments were repeated in the presence of the selective 5-HT serotonin receptor agonists/antagonists. Because SERT is not expressed on astrocytes [58], the experiments were performed considering TDZ agonism at 5-HT_1A_R and its antagonism at 5-HT_2A/C_Rs, α-adrenergic and H_1_ histamine receptors [27, 28]. In contrast, FLUOX, despite the general thought to owe its therapeutic potency to SERT inhibition, has shown relatively high affinity the 5-HT_2B_R in cultured astrocytes [58]; moreover, FLUOX also binds 5-HT_1A/B/D_Rs and antagonises 5-HT_2A/C_Rs and 5-HT_3_Rs [59, 60].

**Fig. 9:**
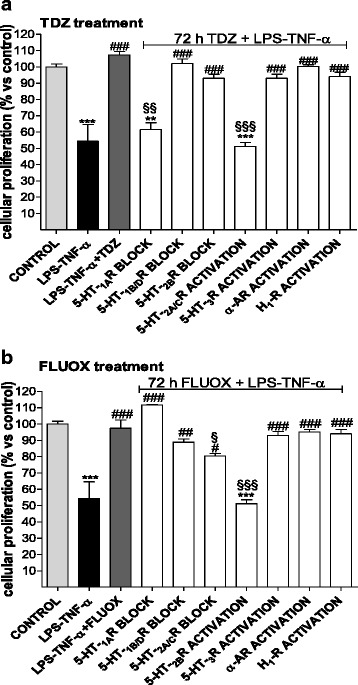
Contribution of 5-HTR receptors in TDZ-elicited cyto-protective effects. **a** Human astrocytes were pre-treated with medium alone (control), or 15 nM (S)-WAY 100135 (5-HT_1A_R antagonist), or 30 nM (R)-DOI, or 250 nM clonidine (α-AR agonist), or 100 μM histamine (H_1_ histamine receptor agonist). After 15 min, cells were incubated with TDZ (10 μM) for 72 h, followed by LPS-TNF-α for an additional 24 h. **b** Human astrocytes were pre-treated with medium alone (control), or 15 nM (S)-WAY 100135 (5-HT_1A_R antagonist), or 10 nM GR 127935 (5-HT_1B/D_R antagonist), or 5 nM RS 127445 (5-HT_2B_R antagonist), or 30 nM (R)-DOI or 100 nM SR 57227 (5-HT_3_R agonist). After 15 min, cells were incubated with FLUOX (10 μM) for 72 h, followed by LPS-TNF-α for an additional 24 h. At the end of treatments, cell proliferation was measured using MTS assay. The data are expressed as percentage with respect to untreated cells (control), set to 100 %, and are the mean ± SEM of three independent experiments, each performed in triplicate. The significance of the differences was determined using a one-way ANOVA-Tukey HSD post hoc test: **P < 0.01, ***P < 0.001 vs. control; ^#^ *P* < 0.05, ^##^ *P* < 0.01, ^###^ *P* < 0.001 vs cells treated with LPS-TNF-α; ^§§^ *P* < 0.01, ^§§§^ *P* < 0.001 vs cells treated with TDZ-LPS-TNF-α (**a**) or FLUOX-LPS-TNF-α (**b**)

The results (Fig. 9a) demonstrated that TDZ failed to reverse LPS-TNF-α-mediated decrease of cell proliferation in the presence of the 5HT-_1A_R antagonist WAY 100135 or of 5-HT_2A/C_R agonist R-DOI. Notably, FLUOX mainly exerted its protective effects through the activation of the 5-HT_2B_R subtype, as demonstrated by the lack of FLUOX-mediated effects in the presence of the selective 5-HT_2B_R antagonist RS-12787445 (Fig. 9b). FLUOX protective effects appeared to be partially mediated by the block of 5HT-_2A/C_Rs (Fig. 9b). Globally, these data demonstrate that the two anti-depressant drugs exert cyto-protective actions involving different 5-HTR subtypes.

### Intracellular pathways associated with TDZ-mediated effects in physiological conditions

The possible intracellular cascades, as the basis of the effects elicited by TDZ, were then investigated. MAPKs have important roles in modulating pro-inflammatory cytokine expression in activated astrocytes and are a common target in glial cells for many of the currently used pharmacological treatments for depression [57, 61–63]. Therefore, the effects of TDZ on the activities of ERK1/2, AKT and JNK in human astrocytes were investigated.

#### ERK 1/2

**Fig. 10:**
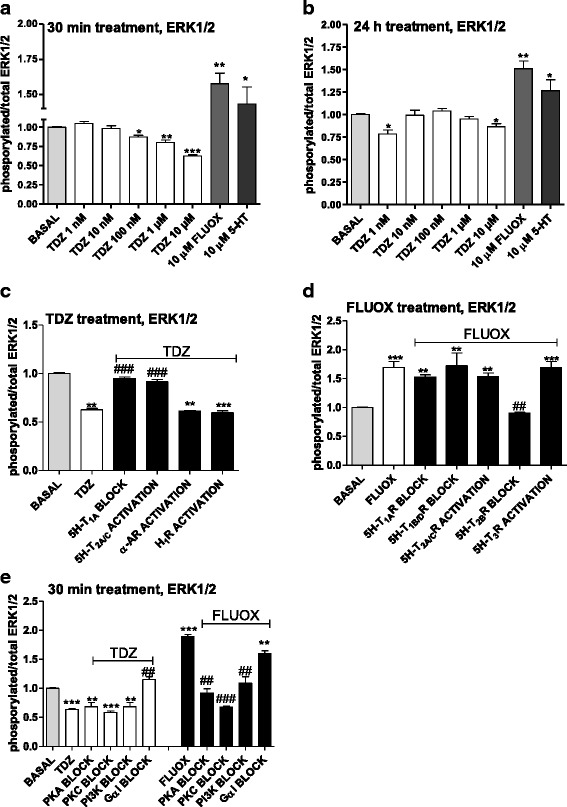
TDZ modulation of the ERK1/2 pathway. **a**, **b** Astrocytes were treated with medium alone (basal), or with the indicated concentrations of TDZ, or FLUOX (10 μM) or 5-HT (10 μM)for 30 min (**a**) or 24 h (**b**). Following incubation, the levels of phosphorylated and total ERK1/2 were evaluated using an ELISA kit as described in the Methods section. The data are expressed as phosphorylated/total ERK1/2 ratio. The data are the mean ± SEM of three independent experiments performed in triplicate. **c** Cells were pre-incubated with medium alone (basal), or 15 nM (S)-WAY 100135 (5-HT_1A_R antagonist), or 30 nM (R)-DOI (5-HT_2A/C_R agonist), or 250 nM clonidine (α-adrenergic receptor agonist), or 100 μM histamine (H_1_ histamine receptor agonist). After 15 min, cells were incubated with TDZ (10 μM) for an additional 30 min. **d** Human astrocytes were pre-treated with medium alone (basal), or 15 nM (S)-WAY 100135 (5-HT_1A_R antagonist), or 10 nM GR 127935 (5-HT_1B/D_R antagonist), or 5 nM RS 127445 (5-HT_2B_R antagonist), or 30 nM (R)-DOI (5-HT_2A/C_R agonist) or 100 nM SR 57227 (5-HT_3_R agonist). After 15 min, cells were incubated with FLUOX (10 μM) for an additional 30 min. Following incubation, the levels of phosphorylated and total ERK1/2 were evaluated using an ELISA kit. **e** Human astrocytes were pre-treated with 200 ng/ml PTX (G_αi/o_ inhibitor), 1 μM H89 (PKA inhibitor), or 1 μM bisindolylmaleimide (PKC inhibitor), or 500 nM wortmannin (PI3K inhibitor); then, cells were incubated with TDZ (10 μM) or FLUOX (10 μM) for an additional 30 min. Following incubation, the levels of phosphorylated and total ERK1/2 were evaluated using an ELISA kit. The significance of the differences was determined using a one-way ANOVA-Tukey HSD post hoc test: *P < 0.05, **P < 0.01 ***P < 0.01 vs. basal; ^##^ *P* < 0.01, ^###^ *P* < 0.001 vs cells stimulated with TDZ or FLUOX

Challenging cells with FLUOX significantly increased p-ERK (Fig. 10a), consistent with previous reports on the SSRI [64]. Surprisingly, TDZ significantly decreased p-ERK/T-ERK ratio in a concentration-dependent manner after 30 min (Fig. 10a). The concentration-dependence was lost in longer treatment (24 h, Fig. 10b), and TDZ significantly decreased basal ERK activation at 1 nM and 10 μM. These data were confirmed by western blot experiments (Additional file 2: Figure S2).

Selected experiments were then performed to shed light on TDZ-receptor targets and second messengers involved in drug modulation of ERK activity.

Pre-treating cells with WAY 100135 or R-DOI counteracted the decrease in ERK phosphorylation elicited by TDZ (Fig. 10c); marginal and not significant effects were noticed in the presence of α-adrenergic agonist clonidine or of histamine (Fig. 10c). These results demonstrate that TDZ-mediated effects on ERKs mainly involved the activation of 5TH_1A_R and the antagonism at 5-HT_2A/C_Rs. In contrast, FLUOX-mediated ERK activation returned to basal value in the presence of the 5HT-_2B_R antagonist RS-127445 (Fig. 10d).

Consistent with 5HT-_2B_R coupling to second messengers, we found that FLUOX-elicited effects on ERK 1/2 were sensitive to cell pre-incubation with PI3K, PKA and PKC inhibitors (Fig. 10e), whereas ERK inhibition by TDZ was blocked in the presence of PTX, thus demonstrating the involvement of Gα_i/o_ proteins (Fig. 10e).

The TDZ-mediated decrease of ERK activity within 24 h of treatment suggests that this anti-depressant activates CREB and BDNF transcription (Fig. 4a) via a different, ERK-independent, mechanism. Consistent with this hypothesis, TDZ enhancement of CREB and BDNF mRNA levels was counteracted by the PI3K inhibitor wortmannin (Additional file 3: Figure S3).

#### AKT

**Fig. 11:**
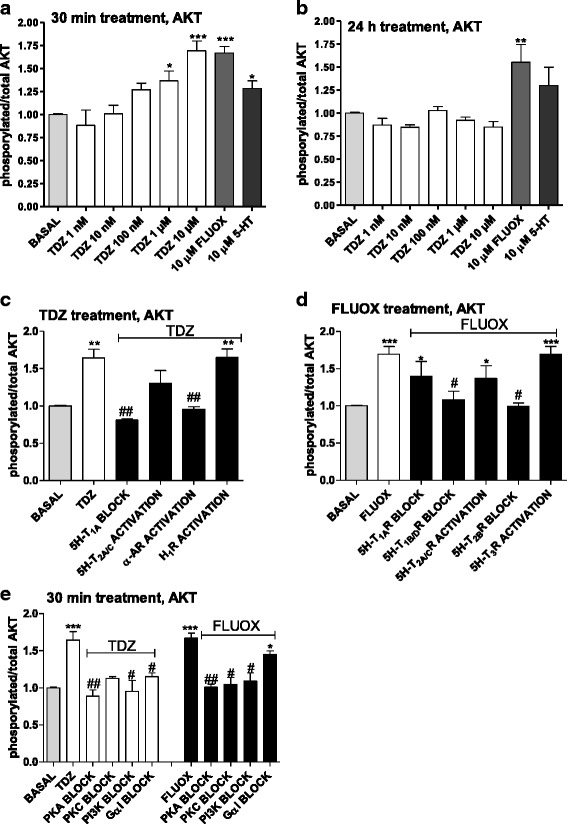
TDZ modulation of the AKT pathway. **a**, **b** Astrocytes were treated with medium alone (basal), or with the indicated concentrations of TDZ, or FLUOX (10 μM) or 5-HT (10 μM) for 30 min (**a**) or 24 h (**b**). Following incubation, the levels of phosphorylated and total AKT were evaluated using an ELISA kit as described in the Methods section. The data are expressed as phosphorylated/total AKT ratio. The data are the mean ± SEM of three independent experiments performed in triplicate. **c** Cells were pre-incubated with medium alone (basal), or 15 nM (S)-WAY 100135 (5-HT_1A_R antagonist), or 30 nM (R)-DOI (5-HT_2A/C_R agonist), or 250 nM clonidine (α-AR agonist), or 100 μM histamine (H_1_ histamine receptor agonist). After 15 min, cells were incubated with TDZ (10 μM) for an additional 30 min. **d** Human astrocytes were pre-treated with medium alone (basal), or 15 nM (S)-WAY 100135 (5-HT_1A_R antagonist), or 10 nM GR 127935 (5-HT_1B/D_R antagonist), or 5 nM RS 127445 (5-HT_2B_R antagonist), or 30 nM (R)-DOI (5-HT_2A/C_R agonist) or 100 nM SR 57227 (5-HT_3_R agonist). After 15 min, cells were incubated with FLUOX (10 μM) for an additional 30 min. Following incubation, the levels of phosphorylated and total AKT were evaluated using an ELISA kit. **e** Human astrocytes were pre-treated with 200 ng/ml PTX (G_αi/o_ inhibitor), or 1 μM H89 (PKA inhibitor), or 1 μM bisindolylmaleimide (PKC inhibitor); then, cells were incubated with TDZ (10 μM) or FLUOX (10 μM) for an additional 30 min. Following incubation, the levels of phosphorylated and total AKT were evaluated using an ELISA kit. The significance of the differences was determined using a one-way ANOVA-Tukey HSD post hoc test: *P < 0.05, **P < 0.01, ***P < 0.001 vs. basal; ^#^ *P* < 0.05, ^##^ *P* < 0.01 vs cells stimulated with TDZ or FLUOX

ELISA and western blot experiments showed that TDZ induced a concentration-dependent significant AKT activation at 30 min of incubation (Fig. 11a, Additional file 4: Figure S4), thus confirming the involvement of PI3K/AKT pathway in TDZ-elicited effects. Phosphorylation was not evidenced in longer treatment (24 h, Fig. 11b). TDZ-mediated AKT phosphorylation appeared to be sensitive to cell pre-incubation with both 5-HT_1A_R antagonist and α-AR agonist (Fig. 11c); moreover, AKT signalling involved Gα_i_ proteins, as well as PI3K and PKA (Fig. 11e).

As a comparison, FLUOX induced AKT activation both after 30 min and 24 h of astrocyte incubation (Fig. 11a, b, Additional file 4: Figure S4), consistent with previous data [65]. FLUOX-elicited effects mainly involved 5HT_2B_R, and in a minor way the 5HT_1B/D_R subtypes (Fig. 11d), depending from PI3K, PKA and PKC signalling proteins (Fig. 11e).

#### JNK

**Fig. 12:**
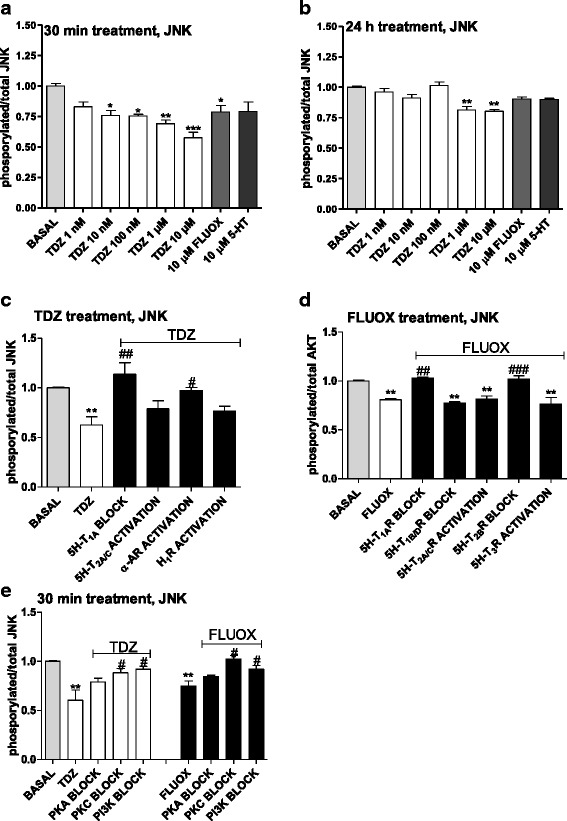
TDZ modulation of the JNK pathway. **a**, **b** Astrocytes were treated with medium alone (basal), or with the indicated concentrations of TDZ, or FLUOX (10 μM) for 30 min (**a**) or 24 h (**b**). Following incubation, the levels of phosphorylated and total JNK were evaluated using an ELISA kit as described in the Methods section. The data are expressed as phosphorylated/total JNK ratio. The data are the mean ± SEM of three independent experiments performed in triplicate. **c** Cells were pre-incubated with medium alone (basal), or 15 nM (S)-WAY 100135 (5-HT_1A_R antagonist), or 30 nM (R)-DOI (5-HT_2A/C_R agonist), or 250 nM clonidine (α-AR agonist), or 100 μM histamine (H_1_ histamine receptor agonist). After 15 min, cells were incubated with TDZ (10 μM) for an additional 30 min. **d** Human astrocytes were pre-treated with medium alone (basal), or 15 nM (S)-WAY 100135 (5-HT_1A_R antagonist), or 10 nM GR 127935 (5-HT_1B/D_R antagonist), or 5 nM RS 127445 (5-HT_2B_R antagonist), or 30 nM (R)-DOI (5-HT_2A/C_R agonist) or 100 nM SR 57227 (5-HT_3_R agonist). After 15 min, cells were incubated with FLUOX (10 μM) for an additional 30 min. Following incubation, the levels of phosphorylated and total JNK were evaluated using an ELISA kit. **e** Human astrocytes were pre-treated with 1 μM H89 (PKA inhibitor), or 1 μM bisindolylmaleimide (PKC inhibitor); after 15 min, cells were incubated with TDZ (10 μM) or FLUOX (10 μM) for an additional 30 min. Following incubation, the levels of phosphorylated and total JNK were evaluated using an ELISA kit. The significance of the differences was determined using a one-way ANOVA-Tukey HSD post hoc test: *P < 0.05, **P < 0.01, ***P < 0.001 vs. basal; ^#^ *P* < 0.05, ^##^ *P* < 0.01, ^###^ *P* < 0.001 vs cells stimulated with TDZ or FLUOX

ELISA assays showed that TDZ or FLUOX alone significantly affected phosphorylated/total JNK levels in astrocytes incubated within 30 min of cell treatment (Fig. 12a). These data were confirmed by western blot (Additional file 5: Figure S5) and globally suggest that JNK inhibition may be involved in the TDZ-induced effects in astrocytes under physiological conditions. The effects were almost completely lost in longer treatment (24 h, Fig. 12b).

The use of selective agonists/antagonists of 5HT-Rs allowed demonstrating that TDZ-mediated effects on JNK activation mainly involved drug activation of 5-TH_1A_R (Fig. 12c). Interestingly, α-AR receptors appeared to contribute to JNK inhibition too (Fig. 12c). In contrast, FLUOX-elicited effects on JNK pathway were mediated by 5-TH_1A_Rs and 5-TH_2B_Rs (Fig. 12d).

JNK inhibition by both FLUOX and TDZ appeared to be sensitive to both PKC and PI3K inhibitors (Fig. 12e).

### Intracellular pathways associated with TDZ-mediated effects under inflammatory insult

**Fig. 13:**
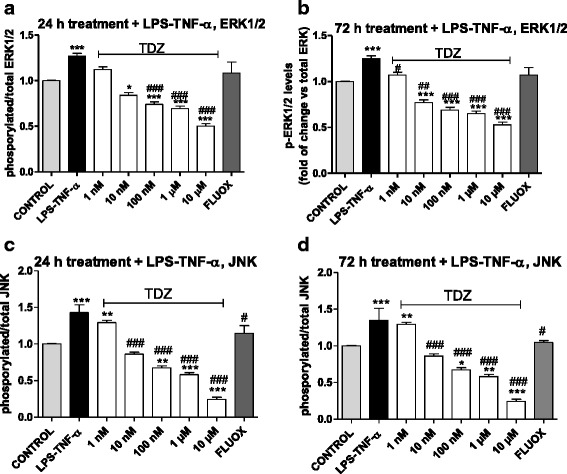
Modulation of the ERK1/2 and JNK pathways in an experimental model of inflammation. Human astrocytes were treated with medium alone (basal), or the indicated concentrations of TDZ, or FLUOX (10 μM) for 24 h (**a**, **c)** or 72 h (**b**, **d**); after drug removal, cells were incubated with LPS-TNF-α for an additional 24 h. Following incubation, levels of phosphorylated and total ERK 1/2 or JNK were evaluated using an ELISA kit as described in the Methods section. The data were calculated as percentages of phosphorylated or total ERK1/2 or JNK relative to untreated cells (basal), which were set at 100 %, and are expressed as phosphorylated/total ERK1/2 (**a**, **b**) or JNK (**c**, **d**) ratio. The data are the mean ± SEM of three independent experiments performed in triplicate. The significance of the differences was determined using a one-way ANOVA-Tukey HSD post hoc test: *P < 0.05, **P < 0.01, ***P < 0.001 vs. control; ^#^ *P* < 0.05, ^##^ *P* < 0.01, ^###^ *P* < 0.001 vs. cells treated with LPS-TNF-α

LPS-TNF-α stimulation enhanced phosphorylated/total ERK (Fig. 13a, b) and JNK (Fig. 13c, d) ratio, thus confirming the latter’s role in astrocyte inflammation [66, 67]. TDZ pre-treatment for 24 h (Fig. 13a) or 72 h (Fig. 13b) still decreased ERK activation. Consistent with these data, following ERK overexpression (Additional file 6: Fig S6 A and B), TDZ-mediated ERK inhibition were completely abolished (Additional file 7: Fig S7).

Moreover, TDZ counteracted the LPS-TNF-α-induced increase in phosphorylated JNK levels after both 24 h (Fig. 13c) and 72 h (Fig. 13d). These results suggest that the protective effects of TDZ against reactive astrocytes at least partially involve the JNK pathway.

## Discussion

In the present paper, TDZ was demonstrated to directly affect human astrocytes in vitro under physiological conditions and following inflammatory insult. TDZ is a triazolopyridine derivative, which is structurally unrelated to other major classes of antidepressants; its molecular and intracellular mechanisms have been investigated in neuronal-like cells and in animal models [29–31]. This is the first study to examine the direct effects of TDZ on astrocytes, both under physiological conditions and inflammation.

Under physiological conditions TDZ significantly up-regulated BDNF and CREB after 24 h, consistent with data obtained with FLUOX or those previously reported for SSRIs in primary cortical astrocyte cultures [25], thus confirming that these factors may contribute to the therapeutic action of anti-depressant drugs also in astrocytes. Surprisingly, a 72-h TDZ challenge significantly increased NF-kB mRNA levels and reduced CREB levels, suggesting astrocytic activation similar to what has been proposed for other antidepressants [68]. Actually, TDZ did not affect astrocyte viability, even after 7 days of incubation. Moreover, TDZ did not modulate the levels of the astrocyte-specific activation marker, GFAP, and did not affect autophagy pathways, thus suggesting that this anti-depressant does not activate astrocytes. Autophagy induction elicited by amitriptyline and the SSRI citalopram [52] was indeed associated with a decrease of neuronal and glial viability.

The effects of TDZ were then tested in an in vitro inflammatory model, established using LPS and TNF-α, which mimics the stress*-*related changes in trophic and pro-inflammatory genes [69, 70]. When astrocytes were pre-treated with TDZ for 72 h before inflammatory insult, cell proliferation and pro- and anti-inflammatory cytokine levels were rescued to control levels, as previously shown in neuronal cells [29] and for other antidepressants, including FLUOX, in glial cells [32, 33]. Moreover, similarly to what observed with FLUOX, TDZ significantly counteracted the LPS-TNF-α-induced mRNA expression of NF-kB, CREB and mTOR. ELISA and western blot analyses confirmed a reduction in NF-kB and CREB activation upon cell pre-treatment with TDZ for 72 h. Consistent with our data, mTOR inhibition has been demonstrated to elicit anti-inflammatory effects in glial cells [47], while beneficial roles of NF-κB inhibition through alteration of the inflammatory environment have been shown [71].

Of note, the effects elicited by TDZ were in many cases not concentration-dependent, probably for its multi-target actions [27, 28]. Additional factors, such as feedback mechanisms, changes in receptor expression and receptor desensitisation/internalisation may occur.

To investigate the contribution of TDZ on astrocyte metabolism, lactate release was analysed. Glucose is metabolized to lactate by astrocytes and is then released into the extracellular space and oxidized by neurons to meet part of their energy needs [56]. In the experimental model of inflammation, LPS-TNF-α significantly decreased the amount of lactate in the culture media; TDZ challenge for 72 h before inflammation induction completely reversed this effect, suggesting that the protective effects of TDZ on activated astrocytes may involve changes in cell metabolism too. Consistent with our data, it has been reported that exposure of murine astrocytes to pro-inflammatory cytokines decreased lactate release in response to glutamate stimulation [72]. Gavillet and colleagues have concluded that a pro-inflammatory environment, such as that present in Alzheimer’s disease or depression, markedly modifies the metabolic phenotype of astrocytes, increasing neuronal vulnerability through disrupted energy metabolism and increased hydrogen peroxide release [72]. In this respect, the increased lactate release by cortical astrocytes in response to FLUOX has been suggested to contribute to protect neurons from stress [73] and to normalize hypometabolism in the prefrontal cortex of depressed patients [73, 74]. Consistent with this hypothesis and with data reported for paroxetine in microglial cells [75], our conditioned media experiments showed that TDZ significantly relieved activated astrocyte-mediated neurotoxicity.

Using selective agonists/antagonists, TDZ was demonstrated to exert its cyto-protective effects through its agonism at 5-HT_1A_Rs and its antagonism at 5-HT_2A/C_Rs. In contrast, FLUOX showed to act through the activation of 5-HT_2B_Rs. SSRI effects exerted via the 5-HT_2B_R had previously been described not only in cultured neurons [76] but also in cultured astrocytes [58]; in particular, this 5-HTR subtype seems to be indispensable for the anti-depressant action of FLUOX [58, 77].

The putative contributions of ERKs, AKT and JNK were then assessed after 30-min and 24-h TDZ treatment, in order to get insight on the receptors involved in the observed effects.

**Fig. 14:**
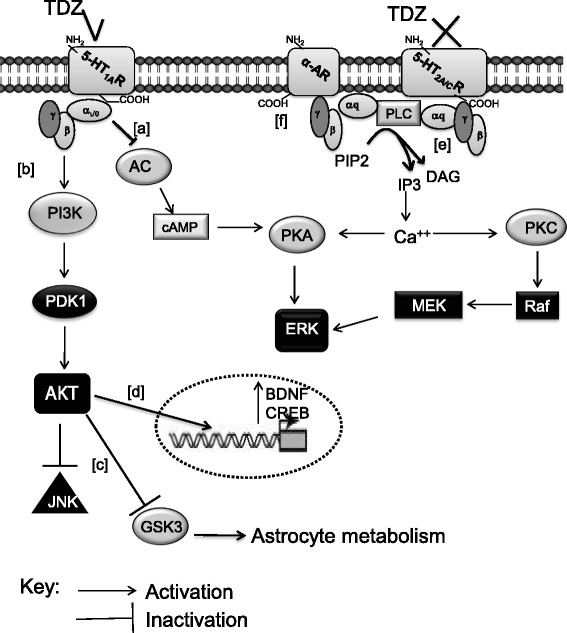
The possible intracellular route of TDZ in human astrocytes. Schematic overview of the possible TDZ/ERK/AKT/JNK signalling pathways in our experimental model is depicted. In astrocytes, the 5-HT_1A_R is coupled to G_αi/o_ proteins [83]; its activation by TDZ decreases ERK phosphorylation via G_αi/o_ protein (*[a]*) [84, 85], and increases AKT phosphorylation (*[b]*) [92, 93]. The PI3K/AKT pathway may contribute to deregulate JNK. Moreover, AKT phosphorylates GSK3, blocking in turn its activity, leading to alteration of astrocyte metabolism (*[c]*) [92, 93]. TDZ enhances CREB and BDNF transcription via a PI3K/AKT pathway (*[d]*). 5-HT_2A/C_Rs may couple to Gα_q_ proteins [40]; TDZ, showing an antagonistic activity on 5HT_2A/C_Rs, may reduce ERK activation (*[e]*). TDZ antagonizes α-ARs (*[f]*), contributing to reduce JNK phosphorylation. Abbreviations: AC: adenylyl cyclase; cAMP: cyclic adenosine monophosphate; PKA: protein kinase A; PKC: protein kinase C; CREB: cAMP response element-binding protein; BDNF: brain-derived nerve factor; ERK: extracellular signal-regulated kinase; PI3K: phosphatidylinositol-4,5-bisphosphate 3-kinase; GSK3: glycogen synthase kinase 3; PDK1: 3-phosphoinositide dependent protein kinase-1; JNKs: c-Jun N-terminal kinases; PLC: Phospholipase C; PIP2: phosphatidylinositol 4,5-bisphosphate; IP3: inositol trisphosphate; DAG: diacyl-glycerol

Consistent with literature data [64, 78], FLUOX significantly activated ERK1/2 by a 5-HT_2B_R-mediated mechanism. Surprisingly, TDZ inhibited basal ERK activity, via activation of 5-HT_1A_Rs and blockage of 5-HT_2A/C_Rs (Fig. 14). 5-HT_1A_Rs couple to inhibitory G-proteins, resulting in decreased cAMP production and PKA activity [79], but the regulation of ERK activity by these receptors in brain is divergent and complicated [80]. Consistent with our finding, some papers have reported 5-HT_1A_R activation decreases ERK phosphorylation in the hippocampus [81–83]. The TDZ-mediated decrease of ERK suggests that this anti-depressant modulates CREB and BDNF transcription via a different, ERK-independent, mechanism. Consistent with this hypothesis, TDZ enhancement of CREB and BDNF mRNA levels was counteracted by a PI3K inhibitor. Similarly, an involvement of PI3K in BDNF promoter activation and in the related CREB transcription has been reported also for other mood stabilizers, such as lithium or valproate [84–86]. Moreover, melatonin has been demonstrated to modulate CREB and GDNF expression in primary astrocytes by a PI3K/AKT mechanism [87].

Consistent with PI3K involvement in TDZ-mediated effects, the drug showed to modulate the downstream effector of PI3K and AKT. Whereas FLUOX activated AKT via 5-HT_2B_Rs transactivation of epidermal growth factor receptor [88], TDZ enhanced AKT phosphorylation mainly involving 5-HT_1A_Rs (Fig. 14). Consistent with our findings, few papers have shown a Gi/o and PI3K sensitive regulation of AKT by 5-HT_1A_Rs [89, 90].

AKT pathway may be involved in TDZ-elicited effects on astrocyte metabolism: as reported for 5-HT and FLUOX [60, 80, 91, 92], activated AKT inhibits glycogensynthasekinase-3(GSK3) [93], thus leading to stimulation of glycogen synthesis and to up-regulation of glucose metabolism. Whereas FLUOX regulation of astrocyte metabolism mainly implicates 5-HT_2B_Rs, an involvement of 5-HT_1A_Rs can be speculated in the case of TDZ, on the light of experimental data showing that 5-HT_1A_Rs have a major role in mediating the AKT-dependent GSK3-regulation [93].

Finally, TDZ, as well as FLUOX, inhibited the constitutive phosphorylation of JNKs (Fig. 14), suggesting that these kinases may function in the effects of anti-depressant under physiological conditions. Consistent with JNK’s role in apoptosis, brain injury and depression [25, 67, 94], when human astrocytes were incubated with LPS-TNF-α, a significant increase in phosphorylated JNK was observed. Similar to the effects of paroxetine in LPS-activated microglia reported by Liu [75], TDZ strongly decreased ERK baseline activity in activated astrocytes.

## Conclusions

In summary, the effect of TDZ on astrocytes under both physiological condition and inflammatory insult has been determined. This study has shown that TDZ alone:

1. decreased the cellular release of the pro-inflammatory cytokine IFN-γ;
2. increased the mRNA expression of neurotrophic and transcription factors;
3. enhanced lactate release;
4. activated AKT mainly through 5-HT_1A_R stimulation;
5. inhibited ERK1/2 and JNK constitutive phosphorylation through 5-HT_1A_R stimulation, 5-HT_2A/C_R blockage or α-AR antagonism.

Most importantly, a pre-treatment with TDZ before the inflammatory insult:

1. completely reversed the decrease of cell proliferation, through a mechanism that involved an activation of 5-HT_1A_Rs and an antagonism at 5-HT_2A/C_Rs;
2. counteracted the decrease of lactate release mediated by LPS-TNF-α;
3. significantly relieved activated astrocyte-mediated neurotoxicity;
4. inhibited inflammation-induced production of inflammatory mediators, such as IL-6 and IFN-γ production in LPS-TNF-α stimulated astrocytes;
5. counteracted the decrease of neurotrophic and transcription factors mediated by LPS-TNF-α after 72 h;
6. counteracted the activation of ERK1/2 and JNK elicited by LPS-TNF-α.

The results at the molecular level, as summarised in the schematic diagram (Fig. 14), demonstrate that TDZ might help normalize trophic and metabolic support to neurons in neuroinflammation, which has been associated with neurological diseases, including major depression. Moreover, these results indicate a potential role for TDZ in neuro-protection via its anti-neuroinflammatory effects in addition to its use in depression.

## Additional files

Additional file 1: Figure S1. Human astrocytes were treated with different concentrations of TDZ for seven days. At the end of treatment, cell proliferation was measured by MTS assay. The data are expressed as percentages relative to untreated cells (control), which were set at 100 %, and represent the mean ± SEM of three independent experiments, each performed in triplicate. Statistical significance was determined using a one-way ANOVA-Tukey post hoc test. (PDF 81 kb) Additional file 2: Figure S2. Human astrocytes were treated with medium alone (basal), or 10 μM TDZ, or 10 μM FLUOX for 30 min. Following incubation, levels of phosphorylated and total ERK 1/2 were evaluated by western blot analysis. (A) Representative western blots. (B) Densitometric analysis of the immunoreactive bands was performed using ImageJ. The data are expressed as the percentage of optical density of the immunoreactive band relative to that of the control, which was set at 100 %, and are the mean values ± SEM of two different experiments. Statistical significance was determined using a one-way ANOVA-Tukey HSD post hoc test: ***P* < 0.01, ****P* < 0.001 vs. control. (PDF 205 kb) Additional file 3: Figure S3. Human astrocytes were pre-treated with 500 nM wortmannin (PI3K inhibitor) or 5 μM PD98059 (MEK1 inhibitor); after 30 min, cells were incubated with TDZ (100 nM) FLUOX (10 μM) for an additional 24 h. At the end of treatment, total RNA was extracted, and relative mRNA quantification of CREB and BDNF was performed by real-time RT-PCR. The data are expressed as fold changes vs. control and represent the mean ± SEM of three different experiments, each performed in duplicate. Statistical significance was determined using a one-way ANOVA-Tukey HSD post hoc test: ***P* < 0.01 vs. control; ^##^ *P* < 0.01 vs cells not treated with the PI3K inhibitor. (PDF 191 kb) Additional file 4: Figure S4. Human astrocytes were treated with medium alone (basal), or 10 μM TDZ, or 10 μM FLUOX for 30 min. Following incubation, levels of phosphorylated and total AKT were evaluated by western blot analysis. (A) Representative western blots. (B) Densitometric analysis of the immunoreactive bands was performed using ImageJ. The data are expressed as the percentage of optical density of the immunoreactive band relative to that of the control, which was set at 100 % and are the mean values ± SEM of two different experiments. Statistical significance was determined using a one-way ANOVA-Tukey HSD post hoc test: ****P* < 0.001 vs. control. (PDF 78 kb) Additional file 5: Figure S5. Human astrocytes were treated with medium alone (basal), or 10 μM TDZ, or 10 μM FLUOX for 30 min. Following incubation, levels of phosphorylated and total JNKs were evaluated by western blot analysis. (A) Representative western blots. (B) Densitometric analysis of the immunoreactive bands was performed using ImageJ. The data are expressed as the percentage of optical density of the immunoreactive band relative to that of the control, which was set at 100 % and are the mean values ± SEM of two different experiments. Statistical significance was determined using a one-way ANOVA-Tukey HSD post hoc test: ****P* < 0.001 vs. control. (PDF 83 kb) Additional file 6: Figure S6. (A,B) Human astrocytes were transfected with an ERK-encoding plasmid. Forty h after transfection, ERK overexpression was verified by western blot, using GAPDH as the loading control. (A) Representative western blots. (B) Densitometric analysis of the immunoreactive bands was performed using ImageJ. The data are expressed as the percentage of optical density of the immunoreactive band relative to that of the control (cells transfected with an empty vector), which was set at 100 %, and are the mean values ± SEM of two different experiments. Statistical significance was determined using a one-way ANOVA-Tukey HSD post hoc test: ****P* < 0.001 vs. control. (PDF 165 kb) Additional file 7: Figure S7. (A,B) Human astrocytes were transfected with an ERK-encoding plasmid. Forty hours after transfection, cells were treated with medium alone (control), different concentrations of TDZ (1 nM-10 μM) for 72 h; after TDZ removal, cells were incubated with 50 μg/ml LPS and 50 ng/ml TNF-α for an additional 24 h. At the end of treatments, cell proliferation was measured using MTS assay. The data are expressed as percentage with respect to untreated cells (control), set to 100 %, and are the mean ± SEM of two independent experiments, each performed in triplicate. The significance of the differences was determined using a one-way ANOVA-Tukey HSD post hoc test: ***P* < 0.01, ****P* < 0.001 vs. control; ^##^ *P* < 0.01, ^###^ *P* < 0.001 vs cells treated with LPS-TNF-α. (PDF 230 kb)

## Abbreviations

BDNF: brain-derived nerve factor
CREB: cAMP response element-binding protein
ERK: extracellular signal-regulated kinase
FLUOX: fluoxetine
GFAP: glial fibrillary acidic protein
GSK3: glycogen synthase kinase 3
HT: serotonin
5-HTRs: 5-HT receptors
JNK: c-jun N-terminal kinase
IL: interleukin
LPS: lipopolysaccharide
MAPK: mitogen-activated protein kinase
mTOR: mammalian target of Rapamycin
NF-κB: nuclear factor κB
PI3K: phosphatidylinositol-4,5-bisphosphate 3-kinase
PTX: pertussin toxin
TDZ: trazodone
TNF-α: tumor necrosis factor alpha
