# Supplementary material for: Trazodone regulates neurotrophic/growth factors, mitogen-activated protein kinases and lactate release in human primary astrocytes
Source: J Neuroinflammation. 2015 Dec 1;12:225. doi: 10.1186/s12974-015-0446-x (PMC4666178; doi:10.1186/s12974-015-0446-x)
Supplement: Additional file 1: Figure S1. — Human astrocytes were treated with different concentrations of TDZ for seven days. At the end of treatment, cell proliferation was measured by MTS assay. The data are expressed as percentages relative to untreated cells (control), which were set at 100 %, and represent the mean ± SEM of three independent experiments, each performed in triplicate. Statistical significance was determined using a one-way ANOVA-Tukey post hoc test. (PDF 81 kb) [file 12974_2015_446_MOESM1_ESM.pdf]

Trazodone regulates neurotrophic/growth factors, mitogen-activated protein kinases and lactate release in human primary astrocytes

Simona Daniele[1#], Elisa Zappelli[1#], Claudia Martini[1*].

[1]Department of Pharmacy, University of Pisa, Italy.

Supplementary Figure 1

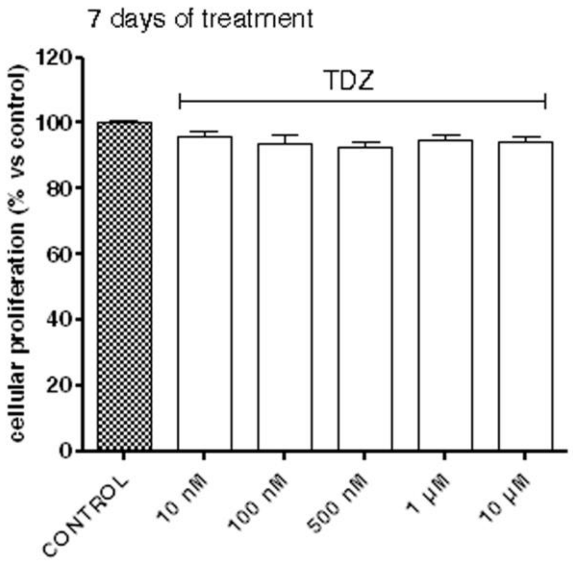

Supplementary Fig. 1. Human astrocytes were treated with different concentrations of TDZ for seven days. At the end of treatment, cell proliferation was measured by MTS assay. The data are expressed as percentages relative to untreated cells (control), which were set at 100%, and represent the mean ± SEM of three independent experiments, each performed in triplicate. Statistical significance was determined using a one-way ANOVA-Tukey post hoc test.
