# Supplementary material for: Trazodone regulates neurotrophic/growth factors, mitogen-activated protein kinases and lactate release in human primary astrocytes
Source: J Neuroinflammation. 2015 Dec 1;12:225. doi: 10.1186/s12974-015-0446-x (PMC4666178; doi:10.1186/s12974-015-0446-x)
Supplement: Additional file 3: Figure S3. — Human astrocytes were pre-treated with 500 nM wortmannin (PI3K inhibitor) or 5 μM PD98059 (MEK1 inhibitor); after 30 min, cells were incubated with TDZ (100 nM) FLUOX (10 μM) for an additional 24 h. At the end of treatment, total RNA was extracted, and relative mRNA quantification of CREB and BDNF was performed by real-time RT-PCR. The data are expressed as fold changes vs. control and represent the mean ± SEM of three different experiments, each performed in duplicate. Statistical significance was determined using a one-way ANOVA-Tukey HSD post hoc test: **P < 0.01 vs. control; ## P < 0.01 vs cells not treated with the PI3K inhibitor. (PDF 191 kb) [file 12974_2015_446_MOESM3_ESM.pdf]

Trazodone regulates neurotrophic/growth factors, mitogen-activated protein kinases and lactate release in human primary astrocytes

Simona Daniele[1#], Elisa Zappelli[1#], Claudia Martini[1*].

[1]Department of Pharmacy, University of Pisa, Italy.

Supplementary Figure 3

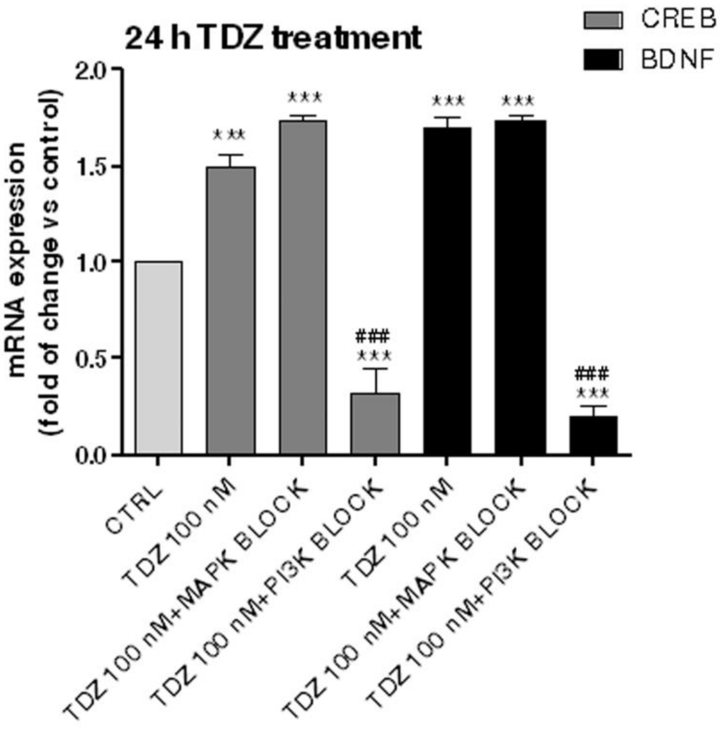

Supplementary Fig. 3. Human astrocytes were pre-treated with 500 nM Wortmannin (PI3K inhibitor) or 5 µM PD98059 (MEK1 inhibitor); after 30 min, cells were incubated with TDZ (100 nM) FLUOX (10 µM) for an additional 24 h. At the end of treatment, total RNA was extracted, and relative mRNA quantification of CREB and BDNF was performed by real-time RT-PCR. The data are expressed as fold changes vs. control and represent the mean ± SEM of three different experiments, each performed in duplicate. Statistical significance was determined using a one-way ANOVA-Tukey HSD post hoc test: **$P<0.01$ vs. control; ##$P<0.01$ vs cells not treated with the PI3K inhibitor.
