# Supplementary material for: Trazodone regulates neurotrophic/growth factors, mitogen-activated protein kinases and lactate release in human primary astrocytes
Source: J Neuroinflammation. 2015 Dec 1;12:225. doi: 10.1186/s12974-015-0446-x (PMC4666178; doi:10.1186/s12974-015-0446-x)
Supplement: Additional file 4: Figure S4. — Human astrocytes were treated with medium alone (basal), or 10 μM TDZ, or 10 μM FLUOX for 30 min. Following incubation, levels of phosphorylated and total AKT were evaluated by western blot analysis. (A) Representative western blots. (B) Densitometric analysis of the immunoreactive bands was performed using ImageJ. The data are expressed as the percentage of optical density of the immunoreactive band relative to that of the control, which was set at 100 % and are the mean values ± SEM of two different experiments. Statistical significance was determined using a one-way ANOVA-Tukey HSD post hoc test: ***P < 0.001 vs. control. (PDF 78 kb) [file 12974_2015_446_MOESM4_ESM.pdf]

Trazodone regulates neurotrophic/growth factors, mitogen-activated protein kinases and lactate release in human primary astrocytes

Simona Daniele[1#], Elisa Zappelli[1#], Claudia Martini[1*].

[1]Department of Pharmacy, University of Pisa, Italy.

Supplementary Figure 4

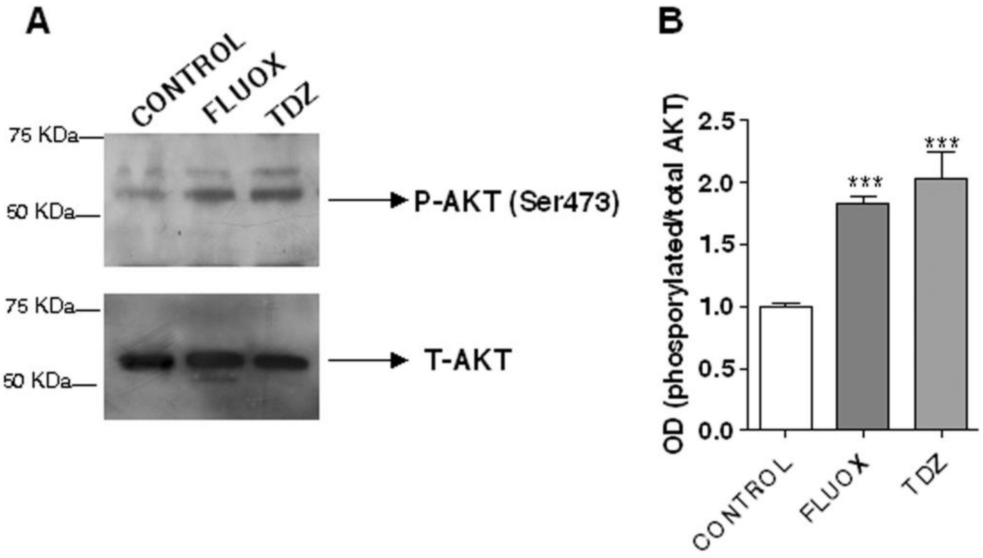

Supplementary Fig. 4. Human astrocytes were treated with medium alone (basal), or 10 µM TDZ, or 10 µM FLUOX for 30 min. Following incubation, levels of phosphorylated and total AKT were evaluated by western blot analysis. (A) Representative western blots. (B) Densitometric analysis of the immunoreactive bands was performed using ImageJ. The data are expressed as the percentage of optical density of the immunoreactive band relative to that of the control, which was set at 100%, and are the mean values ± SEM of two different experiments. Statistical significance was determined using a one-way ANOVA-Tukey HSD post hoc test: ***P<0.001 vs. control.
