# Supplementary material for: Trazodone regulates neurotrophic/growth factors, mitogen-activated protein kinases and lactate release in human primary astrocytes
Source: J Neuroinflammation. 2015 Dec 1;12:225. doi: 10.1186/s12974-015-0446-x (PMC4666178; doi:10.1186/s12974-015-0446-x)
Supplement: Additional file 7: Figure S7. — (A,B) Human astrocytes were transfected with an ERK-encoding plasmid. Forty hours after transfection, cells were treated with medium alone (control), different concentrations of TDZ (1 nM-10 μM) for 72 h; after TDZ removal, cells were incubated with 50 μg/ml LPS and 50 ng/ml TNF-α for an additional 24 h. At the end of treatments, cell proliferation was measured using MTS assay. The data are expressed as percentage with respect to untreated cells (control), set to 100 %, and are the mean ± SEM of two independent experiments, each performed in triplicate. The significance of the differences was determined using a one-way ANOVA-Tukey HSD post hoc test: **P < 0.01, ***P < 0.001 vs. control; ## P < 0.01, ### P < 0.001 vs cells treated with LPS-TNF-α. (PDF 230 kb) [file 12974_2015_446_MOESM7_ESM.pdf]

Trazodone regulates neurotrophic/growth factors, mitogen-activated protein kinases and lactate release in human primary astrocytes

Simona Daniele[1#], Elisa Zappelli[1#], Claudia Martini[1*].

[1]Department of Pharmacy, University of Pisa, Italy.

Supplementary Figure 7

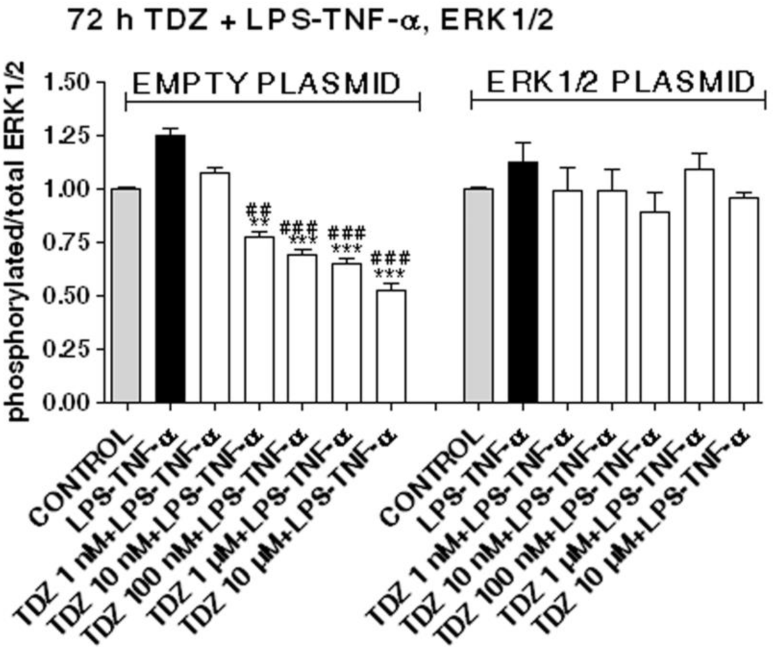

Supplementary Fig. 7. (A,B) Human astrocytes were transfected with an ERK encoding plasmid. Forty h after transfection, cells were treated with medium alone (control), different concentrations of TDZ (1 nM-10 µM) for 72 h; after TDZ removal, cells were incubated with 50 µg/ml LPS and 50 ng/ml TNF-?α for an additional 24 h. At the end of treatments, cell proliferation was measured using MTS assay. The data are expressed as percentage with respect to untreated cells (control), set to 100%, and are the mean ± SEM of two independent experiments, each performed in triplicate. The significance of the differences was determined using a one-way ANOVA-Tukey HSD post hoc test: **P<0.01, ***P<0.001 vs. control; ##P<0.01, ###P<0.001 vs cells treated with LPS-TNF-α.
